# Screening and Mechanistic Investigation of Synergistic Xanthine Oxidase Inhibitors Based on Structure–Activity Relationships

**DOI:** 10.3390/molecules31142485

**Published:** 2026-07-16

**Authors:** Zihan Qu, Xue Zhang, Yuxin Cui, Mengxin Yu, Li Li

**Affiliations:** The College of Chemistry, Changchun Normal University, Changchun 130032, China; 15344364097@163.com (Z.Q.); 15379117126@163.com (X.Z.); 18166814991@163.com (Y.C.); yu13630918072@163.com (M.Y.)

**Keywords:** xanthine oxidase, structure–activity relationship, butein, coreopsin, allopurinol, synergistic inhibition

## Abstract

In order to screen the synergistic inhibitors of xanthine oxidase (XOD), the inhibitory activities of 16 flavonoids in Coreopsis tinctoria were evaluated by structure–activity relationship in this study. Eight flavonoids were screened and were observed to have XOD inhibition and antioxidant capacity, and the zero-interaction potential (ZIP) score was used to determine the synergistic effect with allopurinol. Among them, butein and coreopsin showed good synergistic activity (average ZIP > 15). Subsequently, the inhibitory mechanism of butein and coreopsin alone and in combination with allopurinol on XOD was investigated using in vitro experiments. The inhibitory ability of both on XOD was relatively strong. After combination with two chalcones, the enzyme-inhibition effect of allopurinol was significantly improved. Butein and coreopsin can enhance the binding ability of allopurinol to XOD, induce the conformational change of the enzyme, and achieve synergistic inhibition. The binding mode and key amino-acid binding contribution of the complex were analyzed by computer simulation. In summary, the two chalcones might serve as potential XOD inhibitors and synergistic adjuvants for allopurinol.

## 1. Introduction

XOD mediates the oxidative transformation of hypoxanthine into xanthine, which undergoes subsequent oxidation to uric acid alongside the generation of superoxide anion radicals (O_2_^−^). The accumulation of excess uric acid and reactive oxygen species ultimately triggers hyperuricemia and oxidative injury in tissues [1]. Although allopurinol, as an effective XOD inhibitor, has become a key clinical drug in the treatment of hyperuricemia, it exhibits severe side effects [2]. As suggested by Wen et al. 2024 [3], the screening of natural XOD inhibitors may be a better approach for treating hyperuricemia. Some naturally edible flavonoids have aroused general concerns due to their excellent inhibition of XOD, antioxidant qualities, and scavenging of free radicals. In particular, many flavonoids can not only reduce uric acid production, but can also inhibit the generation of O_2_^−^ catalyzed by XOD, such as galangin, isorhamnetin, epigallocatechin gallate, and gallocatechin gallate [4,5,6,7]. For example, galangin can significantly inhibit XOD and competitively inhibit O_2_^−^ generation in the XOD catalytic process. However, galangin does not show remarkable scavenging activity on DPPH and O_2_^−^ produced from a non-XOD system. Thus, the inhibitory effect of galangin on O_2_^−^ may be achieved through the competitive inhibition of uric acid formation; its mechanism follows a ping-pong mechanism [5].

In particular, combinations of some natural flavonoids may exhibit a very good synergistic effect with allopurinol, reducing the allopurinol amount while achieving stronger inhibitory effects on XOD, such as epigallocatechin gallate, gallocatechin gallate, fisetin, phloretin, baicalein, and baicalin [8,9]. For example, phloretin and allopurinol can bind to different positions of XOD to bring a combined effect by blocking xanthine from entering the Mo center and then impacting electron transfer [3]. These studies suggest that some flavonoids may act as potential co-inhibitors of allopurinol. Some edible flavonoids with a strong inhibition of XOD and combined inhibition with allopurinol may have research potential for hyperuricemia treatment.

*Coreopsis tinctoria* is widely consumed as herbal tea in China [10]. In this study, eight flavonoids with XOD-inhibitory activity were screened from 16 kinds of Coreopsis tinctoria flavonoids (Table 1). The synergistic combinatorial effects of every tested flavonoid with allopurinol were characterized via the ZIP scoring approach. With the average ZIP score > 10 as the screening threshold, butein and coreopsin were finally selected as inhibitors with their excellent synergistic effects (the average ZIP values of both were higher than 15). In order to clarify the internal mechanism of the synergistic inhibition of XOD by the combination of the two and allopurinol, this study constructed the reaction systems of the two chalcone monomers acting alone and synergistically with allopurinol to explore the mechanism. Multispectral analysis showed that the two chalcones alone exhibited XOD-inhibitory activity comparable to allopurinol. In addition, their synergy with allopurinol enhances the interaction with XOD, thereby greatly improving the overall enzyme-inhibition effect of the system. Molecular docking simulations revealed that butein and coreopsin strengthen the binding affinity between allopurinol and XOD. In addition, the binding mode of the complex was analyzed using computer-simulation techniques such as molecular mechanics/Poisson–Boltzmann surface area (mm–PBSA) and the independent-gradient model (IGM); the binding contribution of key amino-acid residues in the binding pocket was quantified. In summary, the in vitro experiments in this study verified that butein and coreopsin might act as potential XOD inhibitors and synergistic adjuvants for allopurinol.

## 2. Results and Discussion

### 2.1. Study on in Vitro Inhibitory Activity and Structure–Activity Relationship

The inhibitory activities of 16 flavonoids in Coreopsis tinctoria against XOD were determined, and the corresponding inhibition rates were computed based on Equation (1).

(1) $$inhibition=(1-\frac{A1-A2}{A3-A2})\times 100\%$$

**Figure 1:**
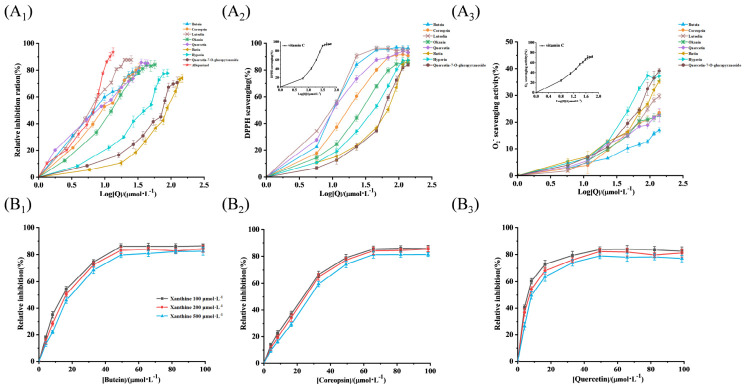
(**A_1_**) XOD-inhibitory effects of 16 flavonoids and allopurinol. (**A_2_**) DPPH scavenging capacity of 16 flavonoids (vitamin C was used as positive control, inset). (**A_3_**) O_2_^−^ scavenging capacity of 16 flavonoids based on the PMS–NADH system (vitamin C was used as positive control, inset). (**B_1_**–**B_3_**) Inhibition of flavonoids 1, 2, and 6 on O_2_^−^ generated by XOD at different [xanthine].

The corresponding IC_50_ values are summarized in Figure 1A_1_ and Table 1. Allopurinol served as the positive reference and exhibited potent XOD-suppressing activity, with an IC_50_ value of 4.16 μmol·L^−1^. This finding is consistent with previously documented data reporting an IC_50_ of 3.81 μmol·L^−1^ [7]. All the tested compounds were divided into three subclasses: chalcones, flavonoids, and flavanones. The significant differences in anti-XOD activity were due to the differences in the planarity of the conjugated skeleton, the substitution position of phenolic hydroxyl groups, and the glycosylation modification. The detailed structure–activity relationship is as follows.

**Figure 2:**
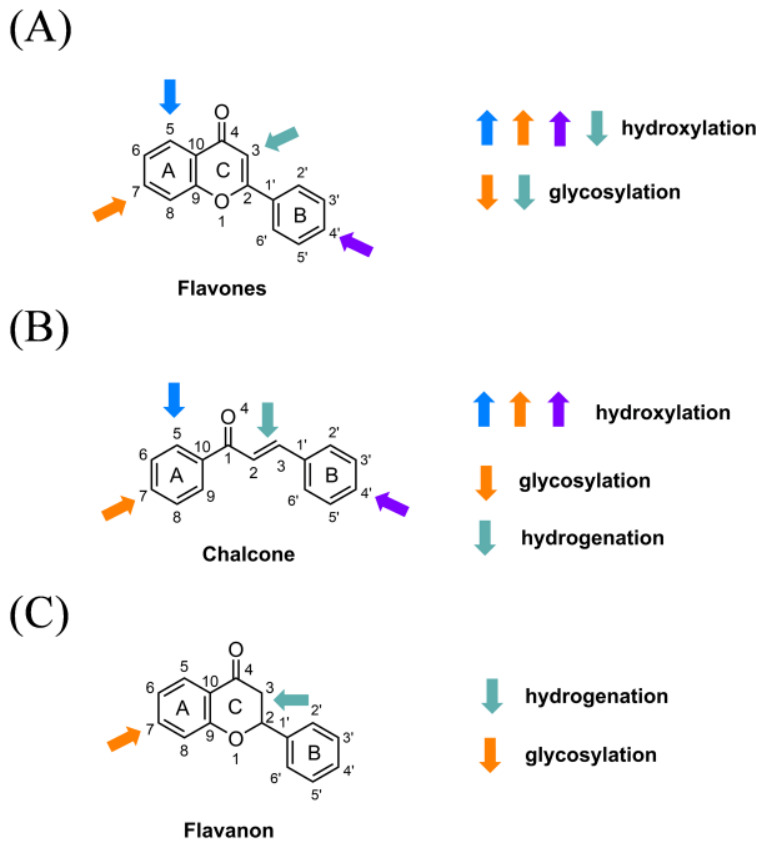
Structural elements affecting the inhibitory effect of (**A**) flavones, (**B**) chalcone, and (**C**) flavanon on XOD. Arrows of different colors represent the structural modification of flavonoids at the corresponding positions of the scaffold. The upward arrow indicates an increase in inhibitory activity, and the downward arrow indicates a decrease in inhibitory activity.

As shown in Figure 2A and Table 1, among the flavonoids, luteolin (5) showed the strongest XOD-inhibitory ability, which was due to the characteristic substitution mode of free hydroxyl groups at C5, C7 of the A ring, and C4′ of the B ring. C5–OH and C7–OH easily form stable hydrogen bonds with polar amino-acid residues in the XOD catalytic pocket, and C4′–OH provides additional intermolecular interactions with the molybdenum cofactor active center. At the same time, its completely coplanar conjugated aromatic skeleton can be tightly hydrophobically stacked with aromatic residues in the binding cavity, jointly endowing luteolin with excellent binding affinity and inhibition efficiency, which is consistent with the structure–activity relationship reported by Zhao et al. 2020 [7]. Compared with luteolin (5), quercetin (6) had a weaker inhibitory effect on XOD, which was attributed to the addition of a C3–OH on its C ring. The hydrophilic C3–hydroxyl group introduced additional polar steric hindrance around the C-ring skeleton, interfered with the planar hydrophobic stacking between the flavonoid skeleton and the XOD pocket residue, and reduced the embedding depth of the ligand in the active cavity, thereby weakening its inhibitory effect.

Glycosylation was identified as an unfavorable structural modification of flavonoid XOD inhibition, consistent with the mechanism clarified by Lin et al., 2015 [11]. The covalently linked sugar group greatly increases the space volume of the molecule, resulting in severe steric hindrance, limiting the ligand from entering the catalytic site of XOD [12]. Corresponding to this rule, the inhibitory activities of three glycosylation flavonoids, hyperoside (11), quercetin-7-*O*-glucopyranoside (9), and rutin (10), were significantly lower than that of aglycone quercetin (6). In particular, marigoldflavin-7-*O*-glucoside (8) completely lost its XOD-inhibitory activity (Figure 2A and Table 1). The co-existence of multiple additional polar hydroxyl substituents and a large glucose group on the A ring produces superimposed polar repulsion and steric hindrance, completely blocking effective ligand–enzyme binding and eliminating detectable inhibitory ability [13].

Among chalcone analogs, butein displays substantially higher XOD-inhibitory potency than coreopsin (Figure 2B). The two compounds share an identical chalcone backbone, with their activity difference stemming from coreopsin’s unique A-ring 7-*O*-glucopyranoside substitution. Butein contains a free B-ring C4′–OH, an essential hydrogen-bond donor stabilizing XOD–ligand complexes [14]. The bulky 7-*O*-glucopyranoside moiety of coreopsin sterically blocks the XOD active pocket entrance and prevents full insertion of the linear chalcone scaffold. Though coreopsin retains the critical C4′ phenolic hydroxyl for protein binding, glycosyl-induced steric hindrance disrupts favorable ligand–protein conformations and drastically reduces the inhibitory efficacy [15]. This comparison further confirms that glycosylation generally attenuates or abolishes the XOD-inhibitory activity of flavonoids and chalcones.

As shown in Figure 2C, in contrast, five flavonoids—naringenin (12), eriodictyol (13), isookanin (14), flavanomarein (15), and taxifolin (16)—showed no XOD inhibition at the tested concentrations, which was due to the saturated single bond at the C2=C3 position of the C ring. The C2=C3 double bond in flavonoids and chalcones supports a fully planar conjugated aromatic skeleton, which mediates strong π–π stacking and hydrophobic interactions between aromatic residues in the binding pocket of XOD [16]. The hydrogenation of C2=C3 in flavanone destroys this planar conjugated system, resulting in a twisted, flexible three-dimensional molecular conformation that cannot match the geometric characteristics of the XOD catalytic cavity [11]. Even with abundant free phenolic hydroxyl groups for hydrogen bonding, the deformed skeleton failed to form a stable binding site with XOD.

Hydroxylation at the C–5 and C–7 positions of the A ring and the C–4′ position of the B ring of flavones and chalcone skeletons improves XOD-inhibitory activity. Glycosylation at the A-ring C–7 attenuates such activity. Furthermore, the multiple polar hydroxyl groups on the A ring and the large glucose portion cause polar repulsion and steric hindrance, thereby reducing inhibitory activity. In addition, hydrogenation at the C2=C3 double bond disrupts the planar conjugated system, which was the main reason for the decrease in the XOD-inhibitory activity of flavanon.

### 2.2. Antioxidant Capacity Analysis

The XOD catalytic process can be defined using a ping-pong mechanism, and the inhibitory mechanism of O_2_^−^ generation can help us more effectively understand the XOD catalytic process [17]. Although flavonoids (1), (2), (3), (5), and (6) have been shown to be potential XOD inhibitors [15], their ability to scavenge XOD O_2_^−^ has been poorly studied. Here, the DPPH radical scavenging, non-enzymatic, and XOD-catalyzed elimination of 8 candidate flavonoids were tested. The data are summarized in Table 2 and Figure 1A_2_,A_3_. Butein (1), luteolin (5), coreopsin (2), and quercetin (6) showed prominent DPPH scavenging activity (IC_50_: 8.00–10.84 μmol·L^−1^), followed by moderate activity of okanin (3) and hyperoside (11). Glycosylated derivatives quercetin–7-*O*-glucopyranoside (9) and rutin (10) showed weaker activity, which was consistent with the fact that glycosylation increased steric hindrance and weakened free-radical capture. Vitamin C was used as a reliable positive control (IC_50_: 14.46 μmol·L^−1^) [18] (Figure 1A_2-inset_). The abundant free phenolic hydroxyl groups give the four flavonoids stronger free-radical blocking ability and protect the acyl chain [19]. In the non-enzymatic O_2_^−^ system, vitamin C achieved a clearance rate of 72.11% at 63.4 μmol·L^−1^ (Figure 1A_3-inset_). At 98.66 μmol·L^−1^, the scavenging rates of 8 flavonoids on hydrogen peroxide were all lower than 50%. (1), (2), (3), and (6) only reached 17.64–23.00%, while glycosylated flavonoids showed slightly better but still unsatisfactory results, revealing the weak direct free-radical scavenging potential of these compounds in chemical systems. In contrast, butein (1), luteolin (5), coreopsin (2), quercetin (6), and okanin (3) showed significant scavenging activity against XOD with IC_50_ values of 5.42–13.38 μmol·L^−1^ (Table 1). These three glycoflavones have weak activity, which can be attributed to the large glycosyl substituents that hinder the binding of the ligand to the XOD active pocket.

In general, butein (1), coreopsin (2), and quercetin (6) simultaneously inhibited XOD activity and XOD-catalyzed production of O_2_^−^, and had strong DPPH scavenging ability. They did not perform well in the enzyme-free O_2_^−^ system, indicating that these flavonoids mainly played a role by inhibiting XOD enzyme activity rather than directly neutralizing superoxide radicals [17]. In addition, the dose-response curves of all 8 flavonoids were almost unaffected by xanthine concentration (Figure 1B_1_–B_3_, Supplementary Figure S1).

### 2.3. Synergistic Inhibition with Allopurinol

The combined effects of 8 flavonoids and allopurinol were quantified by ZIP score (Table 2). The higher ZIP value corresponds to the stronger synergistic effect, and the red area in the heat map represents the synergistic range [20].

The average ZIP scores of butein (1) and coreopsin (2) were the highest, which were 18.19 and 15.52, respectively. As shown in Supplementary Figure S2A, butein and allopurinol had a strong synergistic effect in the range of 1.75–5.6 μmol·L^−1^ and 2–4 μmol·L^−1^, respectively. The synergistic effect of 3.5 μmol·L^−1^ butein and 2.0 μmol·L^−1^ allopurinol was the most significant. For coreopsin, the combination of 5–10 μmol·L^−1^ coreopsin and 3.2–6 μmol·L^−1^ allopurinol showed a significant synergistic inhibitory effect, and the best combination was 8.0 μmol·L^−1^ coreopsin and 3.2 μmol·L^−1^ allopurinol (Supplementary Figure S2B). Similarly, luteolin showed a milder synergistic effect at 3.5–7 μmol·L^−1^ and 2–4 μmol·L^−1^ allopurinol, with an average ZIP score of 8.858 (Supplementary Figure S2C). Okanin had a weak synergistic effect (ZIP = 6.536) (Supplementary Figure S2D), which turned into an antagonistic effect between 26.25 μmol·L^−1^, which was consistent with the concentration-dependent dual effect described by Wen et al. 2024 [3]. The combined inhibitory effect of glycosylation flavonoids (6), (9), and (11) on (Supplementary Figure S2E–H) was negligible, while rutin (10) showed an antagonistic effect with a negative ZIP score.

### 2.4. Study on Inhibition-Interaction Mechanism

After inhibition experimentation, free-radical scavenging experimentation, and ZIP synergistic score, butein (1) and coreopsin (2) not only had a significant inhibitory effect on XOD, but also showed a strong synergistic effect with allopurinol. Subsequently, the binding and inhibition mechanism of XOD was further explored by inhibition kinetic analysis, the multispectral method, molecular dynamics simulation, mm–PBSA calculation, and IGM analysis.

#### 2.4.1. Kinetic Characterization of XOD Inhibition

**Figure 3:**
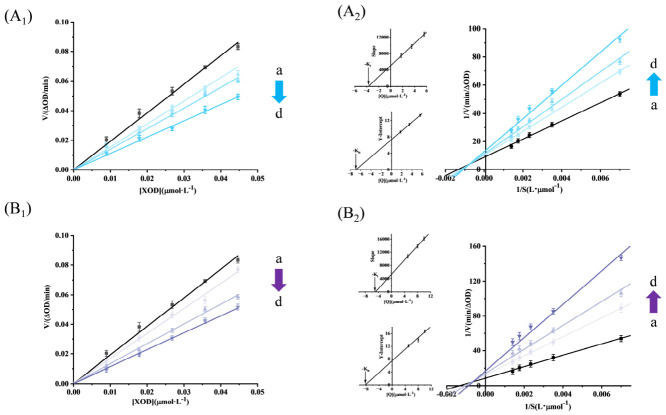
Inhibitory reversibility of butein (**A_1_**,**B_1_**) coreopsin on XOD. Inhibitory type of (**A_2_**) butein and (**B_2_**) coreopsin on XOD. (**A_2-__inset_**) Secondary plots of slope and Y–intercept against [butein]. (**B_2-__inset_**) Secondary plots of slope and Y–intercept against [coreopsin]. Arrows a to d indicate the concentration from low to high.

As shown in Figure 3A_1_,B_1_, at a fixed concentration of XOD, with the increase of chalcones, the enzymatic reaction rate vs XOD showed a good linear relationship, all lines intersecting with the origin, accompanied by a continuous slope decrease. Thus, 2 chalcones reversibly inhibited XOD activity, and two bindings were driven via noncovalent interaction [21]. There are four reversible inhibition types, namely competitive, non-competitive, uncompetitive, and mixed. The inhibitory types of chalcones were determined using Lineweaver–Burk plots. The K_i_ or K_is_ were achieved using Equations (2)–(4). The curve of 1/V vs 1/xanthine in the presence of chalcones is shown in Figure 3A_2_,B_2_. The results showed that the linearity was good, and the 1/V_max_ increased, and 1/K_m_ decreased after the addition of chalcones, which proved that the inhibition of chalcones on XOD followed a mixed mode. Therefore, two chalcones not only bind to XOD but also bind to XOD-xanthine, thereby inhibiting XOD [22]. As shown in Figure 3A_2_,B_2_ inset, K_i_ and K_is_ for XOD–butein 3.71 and 4.92 μmol·L^−1^ and those for XOD–coreopsin were 7.35 and 8.21 μmol·L^−1^, respectively. Based on K_i_ values being smaller than corresponding K_is_, thus, 2 chalcones might bind more easily to XOD alone. The magnitude of the interaction between these two sites can be calculated by α = K_is_/K_i_, and then the mixed inhibitory effect of XOD on chalcones was confirmed again, according to the α value being bigger than 1 [23]. Through the K_i_ value, it was found that butein had a stronger effect on XOD than coreopsin, which verified the IC_50_ conclusion.

(2) $$\frac{I}{V}=\frac{K_{m}}{V_{max}}\left(1+\frac{\left[Q\right]}{K_{i}}\right)\frac{1}{\left[s\right]}+\frac{1}{V_{max}}\left(1+\frac{\left[Q\right]}{{\alpha K}_{i}}\right)$$

(3) $$slope=\frac{K_{m}}{V_{max}}+\frac{K_{m}[Q]}{V_{max}K_{i}}$$

(4) $$Y-intercept=\frac{1}{V_{max}}+\frac{1}{K_{is}V_{max}}[Q]$$

In Equations (2)–(4), K_m_, V, V_max_, [Q], and [S] show the Mi constant, initial reaction speed, maximum reacted speed, chalcones, and xanthine concentration, respectively.

#### 2.4.2. Intermolecular Interaction Analyzed Using Fluorescence

**Figure 4:**
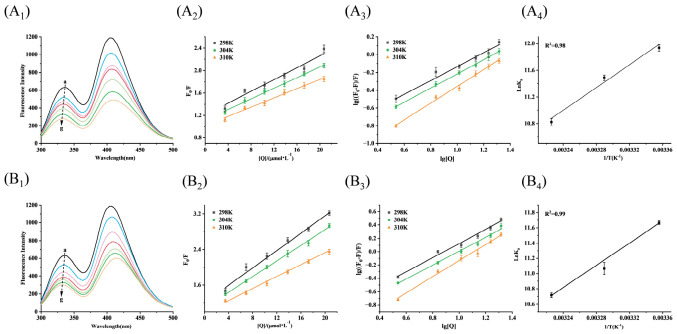
Fluorescence spectra, Stern–Volmer graphs, log(F_0_−F)/F versus log[chalcone], and Van’t Hoff plot. (**A_1_**–**A_4_**) XOD–butein, (**B_1_**–**B_4_**) XOD–coreopsin.

XOD generates endogenous fluorescence since the presence of Trp, Tyr, and Phe moieties, as well as the interaction between XOD and small active molecules, can be investigated by monitoring fluorescence changes before and after its binding to small molecules [24]. Equation (5) is used to remove the inner filter effects. Then, quenching analysis can be carried out to examine the interaction patterns of XOD and 2 chalcones. In Figure 4A_1_,B_1_, two main peaks at 335.8 and 406.2 nm of XOD were observed, while 2 chalcones had no peak there. With the successive addition of chalcones, XOD intensity continuously decreased, indicating that 2 chalcones could interact with the amino-acid residue in XOD [25]. When 2 chalcones were 20.7 μmol·L^−1^, butein and coreopsin caused a 55.7 and 52.29% quenching rate, accompanied by an obvious blue shift. Based on the fact that butein caused a greater blue shift (6 nm) than coreopsin (4 nm), it was determined that butein enhanced XOD hydrophobicity more significantly [26].

The fluorescence-quenching interaction of active material with large molecules is divided into dynamic, static, or mixed quenching [27]. As shown in Figure 4A_2_, quenching of butein to XOD was determined using a single quenching type according to a favorable linear relationship of F_0_/F vs [butein]. Similarly, coreopsin also showed the same quenching type. (Figure 4B_2_) [28]. Furthermore, K_SV_ and K_q_ obtained by Equation (6) were used to evaluate the quenching mechanism of 2 chalcones on XOD. With the increase of experimental temperature, the relevant K_SV_ of chalcones decreased, and the relative K_q_ were greatly bigger than 2.0 × 10^10^ L/mol·s. Therefore, 2 chalcones quenched XOD fluorescence mainly following the static quenching mechanism (Table 3), and formation of XOD–chalcones led to a decrease in XOD peak [29]. The binding constant (K_a_) and the number of binding sites (n) were calculated by Equation (7) [30], and the results are shown in Figure 4A_3_,B_3_. At three temperatures, butein had a stronger affinity with XOD than coreopsin based on K_a_ values listed in Table 3. These results confirmed that XOD could form a more stable complex with butein than with coreopsin, leading to greater quenching of fluorescence [31]. At 298 K, the Ka value of the XOD–butein complex reached 12.22 × 10^5^ L·mol^−1^, and the Ka value of the XOD–coreopsin complex was 11.64 × 10^5^ L·mol^−1^. Both of them had strong binding affinity to XOD, but butein showed stronger binding effect [32]. By increasing the temperature, the K_a_ value of the two complexes decreased, indicating that low temperature was more conducive to complex stability. ΔS, ΔG, and ΔH obtained via Equations (8) and (9) are listed in Table 3. Spontaneous binding processes of 2 chalcones with XOD were determined using -ΔG. Both -ΔH and -ΔS proved that the three 2-chalcones-XOD were mainly dominated by the hydrogen bond and van der Waals force [33].

(5) $$F_{cor}=F_{obs}\times 10^{\left(A_{ex}+A_{em}\right)/2}$$

(6) $$\frac{F_{0}}{F}=1+K_{sv}[Q]={1+K_{q}\tau }_{0}[Q]$$

(7) $$\log\frac{F_{0}-F}{F}=\log K_{a}+\mathrm{nlog}[Q]$$

(8) $$\ln K_{a}=-\frac{\Delta H}{RT}+\frac{\Delta S}{R}$$

(9) ΔG=ΔH − TΔS

Here, F_cor_/F_obs_ show corrected/uncorrected fluorescence; A_ex_ and A_em_ indicate chalcone absorbance at λ_ex_ and λ_em_; F_0_ and F indicate fluorescence for XOD and XOD-excessed chalcones; [Q] indicates chalcone amounts; R refers to the gas constant.

#### 2.4.3. Conformation Changes in XOD Induced by Intermolecular Interactions

According to thermodynamic characterization, the two chalcones bind XOD predominantly through hydrogen bonding and van der Waals forces. To further clarify the protein structural alterations triggered by the two ligands, we adopted a combined strategy of spectroscopic detection and theoretical simulation: synchronous fluorescence, UV–Vis, 3D fluorescence, and FT–IR spectra were measured experimentally, while molecular dynamics, MM–PBSA, and IGM calculations were carried out for auxiliary analysis.

Synchronous fluorescence spectroscopy serves as a powerful technique to characterize microenvironmental variations of amino-acid residues in proteins [34]. In particular, synchronous signals corresponding to Tyr and Trp residues can be separately monitored by setting Δλ to 15 nm and 60 nm, respectively. As shown in Supplementary Figure S3A_1_, no obvious wavelength shift was detected for the peak measured at Δλ = 15 nm. In contrast, a distinct blue shift was observed in the synchronous fluorescence peak at Δλ = 60 nm upon the addition of butein (Supplementary Figure S3A_2_), which confirmed the formation of the butein–XOD complex and indicated a notable increase in the hydrophobicity around Trp residues, demonstrating that butein barely disturbed the hydrophobic micro-environment of Tyr residues [21]. To further quantify the individual quenching contributions of Tyr and Trp to the intrinsic fluorescence of XOD, relative synchronous fluorescence quenching (RSFQ) values were calculated (Supplementary Figure S3A_3_). The RSFQ value of Trp reached 58.64%, which was remarkably higher than the value of Tyr (48.78%) at identical butein concentrations, revealing that Trp residues dominated the overall fluorescence-quenching process of XOD [35]. Comparable spectral variations were also observed when XOD was incubated with coreopsin (Supplementary Figure S3B_1_–B_3_).

The micro-environment and conformation changes of fluorescent amino acid in XOD can be intuitively reflected by 3D fluorescence [36]. In Supplementary Figure S3C_1_–C_3_, peak a was the Rayleigh scattering peak, and peaks 1 and 2 were the Trp and Tyr peaks, respectively. Peaks 3 and 4 were relative to the peptide skeleton of XOD [37]. Furthermore, peaks 1 and 2 of XOD decreased clearly in the presence of butein, suggesting that butein could react with Tyr and Trp. Meanwhile, a significant blue shift of peak 1 appeared, indicating that hydrophobicity near Trp was enhanced by butein [17]. Peaks 3 and 4 also showed a significant decrease with a blue shift. Similar results also appeared in the effects of coreopsin on 3D spectra of XOD, but the decrease and blue shift of butein were stronger than that of coreopsin. The 3D experimental results further verified fluorescence and synchronous results.

**Figure 5:**
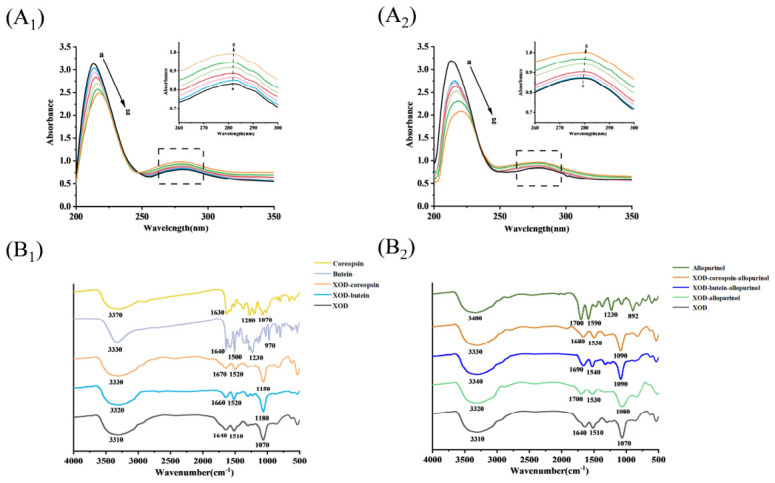
UV spectra of XOD without and with (**A_1_**) butein and (**A_2_**) coreopsin. FT–IR spectra of XOD with chalcones (**B_1_**), and XOD with allopurinol in the absence and presence of chalcones (**B_2_**).

Peak position changes and hyperchromic and hypochromic impacts of characteristic peaks can reflect the micro-environment of proteins and complexes formation in the UV–vis spectra. The UV–vis spectra of chalcones are depicted in Figure 5A. Two major bands are near 210 and 280 nm, distributing to π→π* transitions from C=O on the peptide bond and aromatic residues of XOD, respectively [37]. Upon adding butein to XOD, the band at 210 nm revealed a hypochromic effect with a pronounced red shift, implying that XOD conformation was altered by butein (Figure 5A_1_). However, the peak at 280 nm increased slowly with red shift (color enhancement effect), proving that butein formed a complex with XOD [38]. Similar UV results also appeared in the impact produced by coreopsin (Figure 5A_2_). Direct change in UV–vis spectra confirmed that XOD was statically quenched by two chalcones, which supported the results of the fluorescence-quenching experiment.

FT–IR spectral measurements were performed to characterize chalcone conformational variations of XOD, and the corresponding data are illustrated in Figure 5B_1_. Two chalcones exhibited some unique bands in FT–IR spectra. For example, a broad and prominent band was observed at 3330 cm^−1^, attributed to O–H stretching in chalcones. Bands at 1640 cm^−1^ came from C–H bonds and C=O bonds stretching vibration. The bands (1500 and 1230 cm^−1^) were made via the C–O stretching vibration. Two chalcones belong to flavonoids, and some characteristic bands were similar to FT–IR bands of rutin reported by Paczkowska et al. 2015 [39].

FT–IR amide band shifts are tightly correlated with protein-conformational states [40]. In Figure 5B_1_, A broad amide A band centered at 3310 cm^−1^ was observed in free XOD, which originated from N–H or O–H stretching vibrations [41]. For XOD combined with butein and coreopsin, the characteristic peaks moved to 3320 cm^−1^ and 3330 cm^−1^, respectively. This spectral shift demonstrates hydroxyl-mediated hydrogen-bond interactions between the two compounds and XOD [42], agreeing with partial thermodynamics results. As the band is highly responsive to secondary structural alterations, Amide I has drawn extensive research attention. Interactions between active compounds and protein molecules often alter Amide I’s peak intensity and wavenumber position [43]. The band at 1640 cm^−1^ belonged to the Amide I band (Figure 5B_1_). After XOD combined with butein and coreopsin, the Amide I peaks shifted to 1660 cm^−1^ and 1670 cm^−1^, respectively. The native XOD signal at 1510 cm^−1^ was assigned to Amide II, which moved to 1520 cm^−1^ for both ligand–protein complexes. These spectral variations collectively verify that the two chalcones induce conformational rearrangements of XOD [44]. Amide I peak deconvolution was carried out to characterize alterations in XOD secondary structure, and all calculated parameters are presented in Table 4. XOD contained 24.39% α–helix, 46.22% β–sheet, 11.56% β–turn, 16.61% random coil, and 1.21% random coil. It could be seen that the β–sheet was the secondary structure with the highest content, followed by α–helix, random coil, β–turn and β–antiparallel. When complexed with butein, XOD showed reduced proportions of α–helix, β–sheet, and β–turn, alongside elevated random coil and antiparallel β–sheet contents. The influence of coreopsin on XOD secondary structure also showed a similar rule. Based on the reduction in α–helix and β–turn, thus, two chalcones destroyed the hydrogen bonds of XOD, causing the β–sheet structure to open, resulting in the XOD structure becoming loose and finally inhibiting XOD activity [34].

#### 2.4.4. Dynamics Simulation Caused by Intermolecular Interactions

**Figure 6:**
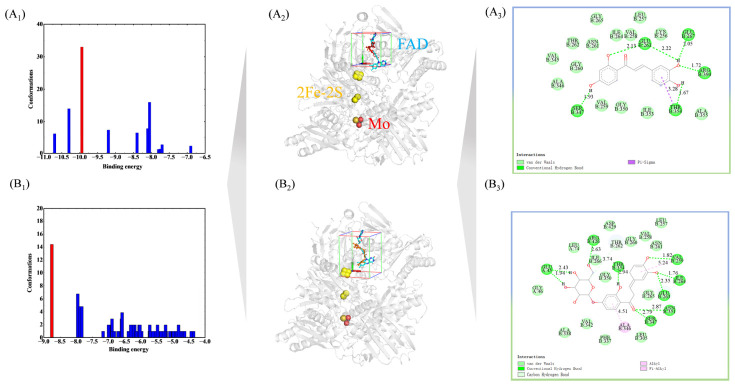
Binding-energy distribution of different docking conformations for the XOD–butein (**A_1_**) and XOD–coreopsin (**B_1_**). 3D overall binding conformation of butein (**A_2_**) and coreopsin (**B_2_**) within the active pocket of XOD. 2D schematic diagram of intermolecular interactions between butein (**A_3_**)/ coreopsin (**B_3_**) and amino-acid residues of XOD.

The docking results with the smallest energy in the highest conformation were chosen (Figure 6). Docking energies of XOD–allopurinol, XOD–butein, and XOD–coreopsin were −10.06, −9.94, and −8.78 kcal/mol, respectively (Figure 6A_1_), suggesting that interacting processes were spontaneous. Docking energy data revealed that allopurinol forms the most stable complex with XOD and delivers the optimal inhibitory capacity, with butein and coreopsin exhibiting weaker effects in sequence [45]. This verified the IC_50_ conclusion from the perspective of theoretical docking. As revealed in Figure 6A_3_, butein generated hydrogen bonds with GLU263 (2.13 Å, 2.22 Å), THR354 (1.67 Å), ARG394 (1.72 Å), SER347 (1.93 Å), and GLU267 (2.05 Å). Coreopsin produced hydrogen bonds with GLU45 (1.94 Å, 2.43 Å), ILE264 (1.76 Å), VAL259 (1.82 Å), GLU263 (2.35 Å), ARG426 (2.63 Å), SER347 (2.79 Å), ASN351 (2.87 Å), and THR354 (2.94 Å) (Figure 6B_3_). GLU45, GLU263, ARG394, and ARG426 were considered to be key amino acids in FAD, indicating that 2 chalcones could bind with FAD [31,46] (Figure 6A_2_,B_2_). Meanwhile, 2 chalcones were also surrounded by some residues, forming van der Waals forces. In addition, some noncovalent interactions also participated in the formation of the 2 complexes.

**Figure 7:**
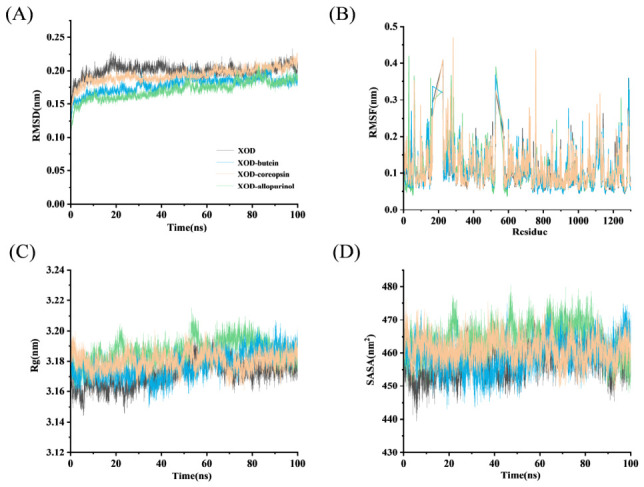
Dynamics diagram. (**A**) RMSD, (**B**) RMSF, (**C**) Rg, (**D**) SASA.

Dynamics simulation further introduced allopurinol as a positive control to compare the binding mode, which laid a foundation for the later exploration of its synergistic effect. Key parameters extracted from molecular dynamics simulations facilitate insight into the microscopic binding process between ligands and XOD protein [34]. Similarly, these parameters are used to evaluate protein–ligand stability [47]. Dynamic characteristics of XOD were analyzed under two conditions: with and without the addition of chalcones. RMSD values were calculated to quantify structural departures from the initial XOD conformation, so as to further compare the structural stability of XOD–chalcone and XOD–allopurinol complexes. In Figure 7A, the RMSD for two XOD–chalcones differed from XOD, exhibiting equilibrium with smaller RMSD values than XOD. Therefore, chalcones could bind to XOD through some noncovalent interactions, which changed the original conformation, making its conformation more stable and playing an inhibitory role [48]. RMSF fluctuation degree commonly reflects the residue flexibility [49]. In Figure 7B, the RMSF values between XOD and XOD–butein were different, and some residues showed obvious fluctuations. For example, some residue flexibility was enhanced after binding to butein since some RMSF values of XOD–butein were higher than those of XOD alone. By contrast, some residues of XOD have lower RMSF values than those of XOD–butein complex, indicating that the freedom of these residues was reduced by butein. Similar phenomena also occurred in RMSF values during binding with coreopsin and allopurinol. Herein, the binding of chalcones and allopurinol caused fluctuations of these residues and further stabilized complexes by adjusting their conformations. Rg represents the average distance between the center and edge of the enzyme over a period, and it can usually be used to examine overall enzyme-inhibitor compactness [17,24]. SASA values reflect the surface changes of the system in contact with a solvent [47]. The lower Rg and SASA values usually indicate a more compact system, while higher values indicate a looser system. In Figure 7C, the Rg of XOD fluctuated during 0–30 ns, and stabilized at about 3.17 nm. Compared to XOD, Rg values for XOD–butein were relatively stable during the whole simulation. Rg for XOD–coreopsin and XOD–allopurinol fluctuated obviously and then gradually stabilized after 50 ns. Three complex Rg values were obviously higher than XOD, illustrating that the interaction of XOD and chalcones/allopurinol made the XOD structure looser [22]. Figure 7D depicts the SASA value changes between the XOD and chalcones. The SASA values of XOD fluctuated evidently and finally plateaued at approximately 457.39 nm^2^. Compared with XOD itself, the three complexes have larger SASA values, indicating that XOD makes its conformation looser during the binding process [17].

#### 2.4.5. Elaboration on Complex Stability Based on Binding Free Energy

**Figure 8:**
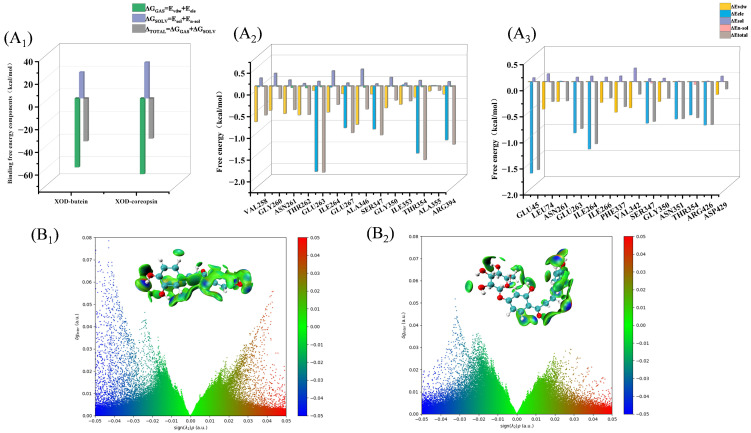
(**A_1_**) ΔG_GAS_, ΔG_SOLV_, and Δ_TOTAL_ values, (**A_2_**) Energy decomposition of residue of XOD mediated by butein and (**A_3_**) coreopsin. IGM of XOD–butein (**B_1_**) and XOD–coreopsin (**B_2_**).

As presented in Figure 8A_1_, MM–PBSA calculations were implemented to compute ΔG_GAS_, ΔG_SOLV_, and total binding free energy, ΔGtotal [50]. Electrostatic and van der Waals energies were crucial energies that participated in the formation of two complexes since ΔG_GAS_ values were significantly lower than corresponding ΔG_SOLV_ [51]. Moreover, Δ_TOTAL_ of XOD–butein and XOD–coreopsin were −37.80 and −35.05 kcal/mol, affirming that binding of chalcones to XOD was spontaneous [52]. These also verified docking results from a dynamics perspective; the XOD–butein complex was more stable than XOD–coreopsin. Amino-acid energy decomposition helps clarify the individual residue contributions to ligand–protein complex assembly. As confirmed by several studies, the residues with larger negative decomposition binding free energies might contribute more to complex formation [53]. In Figure 8A_2_, in butein–XOD formation, GLU263 greatly supplied electrostatic energy, mainly by hydrogen bond. Meanwhile, GLU267, SER347, THR354, and ARG394 provided more electrostatic energy as well. In the binding of coreopsin to XOD, GLU45, VAL259, GLU263, ILE264, SER347, ASN351, THR354, and ARG426 also contributed more electrostatic energy (Figure 8A_3_). It was worth mentioning that some amino acids also greatly contributed to maintaining the stability of the 2 complexes by giving enough van der Waals energy (Figure 8A_2_,A_3_). Thus, these two energies made an indelible contribution to the formation and stability of the 2 complexes, which verified the docking conclusion.

#### 2.4.6. Visualization of Noncovalent Interactions Using IGM

IGM, built upon electron density distribution, enables intuitive visualization of both covalent and noncovalent intermolecular contacts [20]. The IGM approach adopts color-coded mapping to intuitively distinguish different types and intensities of intermolecular interactions [54]. The δg_inter_ index offers a more precise reflection of intermolecular contacts. Higher color saturation corresponds to stronger intermolecular binding forces. Figure 8B_1_,B_2_ showed several observable noncovalent interactions of XOD with chalcones, which were mainly expressed as regions of weak to moderate attraction, including some regions. Usually, dark blue and light blue regions correspond to strong and medium hydrogen bonds, respectively. Butein had a stronger hydrogen-bond interaction than coreopsin due to it exhibiting higher δg_inter_ values in dark blue regions [55]. Deep green regions indicate strong van der Waals contacts, and pale green parts represent weak ones. Rich green domains exist on the binding surfaces between XOD and both chalcones, demonstrating that van der Waals forces participate in maintaining the 2 complexes [56]. Sparse red domains could be observed as well, which demonstrates that weak spatial repulsion occurs during the formation of the 2 complexes [56]. The δg_inter_ isosurfaces at isovalues of 0.006 for XOD–chalcones are inserted in Figure 8B_1_,B_2_, and color converted from blue, green to red. There were some dark and light blue regions in Figure 8B_1_,B_2 inset_, revealing that stronger hydrogen-bond interactions existed in two complexes [57]. Furthermore, both chalcones are enveloped by extensive green regions, further verifying that van der Waals interactions play a vital role in stabilizing the complexes [57]. There were also a few red regions in Figure 8B_1_,B_2 inset_, indicating the presence of a smaller steric effect [58]. Consistent with fluorescence quenching, molecular docking, and MM–PBSA results, IGM analysis offered additional supporting evidence.

### 2.5. Synergistic Inhibitory Effect of Chalcones and Allopurinol

#### 2.5.1. Synergistic Inhibition Kinetics

**Figure 9:**
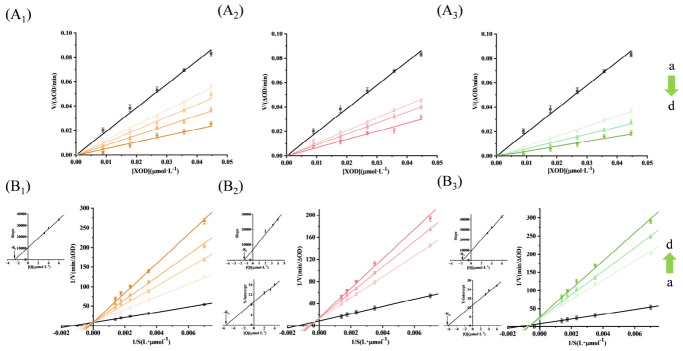
Inhibitory reversibility with allopurinol (**A_1_**), allopurinol–butein (**A_2_**), and allopurinol–coreopsin (**A_3_**), and inhibitory type (**B_1_**–**B_3_**). (**B_inset_**) Secondary plots of slope and Y–intercept against [allopurinol\allopurinol–chalcone]. Arrows a to d indicate the concentration from low to high.

As shown in the ZIP screening experiment, butein and coreopsin displayed a significant combined effect with allopurinol. To study the combined inhibitory kinetics behavior of chalcones with allopurinol, in the non-existence and existence of chalcones, the reversibility of allopurinol on XOD was measured. At a fixed xanthine, with the continuous increase of XOD and allopurinol, the reaction displayed a linear relationship with allopurinol, intersecting with the origin, and corresponding slopes indicated a downward trend (Figure 9A_1_). Compared with allopurinol alone, the presence of two chalcones resulted in a greater decrease in the slope at the same allopurinol concentration (Figure 9A_2_,A_3_). This indicates that allopurinol is a reversible inhibition in the absence and presence of two chalcones. Since the straight line intersects with the y–axis at one point (Figure 9B_1_), it is judged that allopurinol is a competitive inhibition type [3]. On the contrary, in the presence of two chalcones, allopurinol was a mixed-type inhibitor (Figure 9B_2_,B_3_). Therefore, in the presence of chalcones, allopurinol competes with xanthine for active sites [3]. The Ki values of allopurinol were 1.32 and 1.69 μmol·L^−1^ in the presence of butein and coreopsin, respectively, which were lower than those of allopurinol alone (2.80 μmol·L^−1^). Therefore, the presence of two chalcones enhanced the inhibitory effect of allopurinol on XOD [59].

#### 2.5.2. Synergistic Inhibition of Fluorescence and Synchronous Spectral Analysis

**Figure 10:**
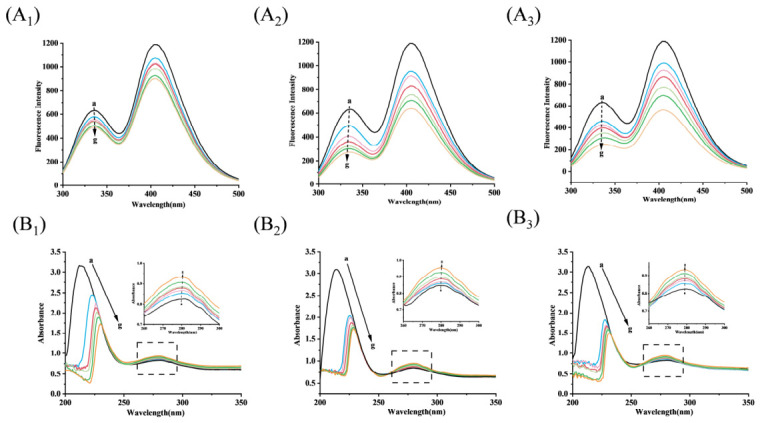
(**A_1_**) Fluorescence spectra of XOD induced by allopurinol, (**A_2_**) allopurinol–butein, (**A_3_**) allopurinol–coreopsin. (**B_1_**) UV spectra of XOD changed by allopurinol, (**B_2_**) allopurinol–butein, and (**B_3_**) allopurinol–coreopsin.

**Figure 11:**
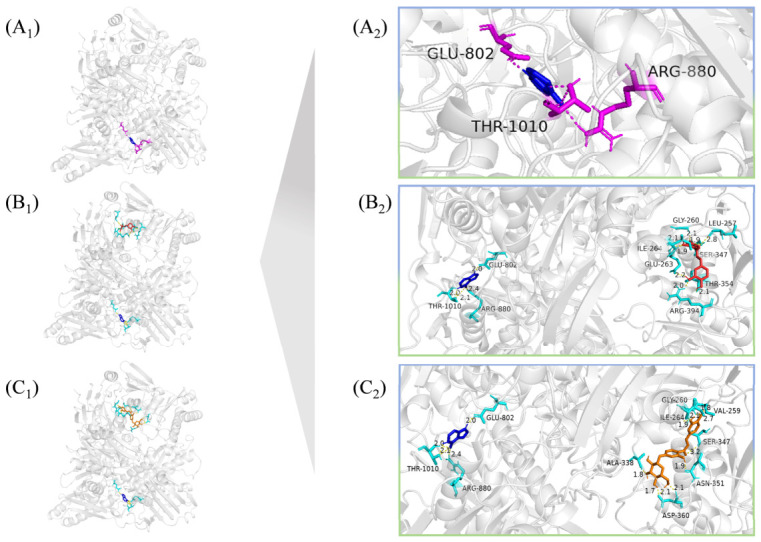
3D overall binding conformation of allopurinol (**A_1_**), allopurinol–butein (**B_1_**), and allopurinol–coreopsin (**C_1_**) within the active pocket of XOD. 2D schematic diagram of intermolecular interactions between allopurinol (**A_2_**)/allopurinol–butein (**B_2_**)/allopurinol–coreopsin (**C_2_**) and amino-acid residues of XOD.

As shown in Figure 10A_1_, allopurinol exhibits a negligible fluorescence-quenching effect toward XOD. Even with elevated allopurinol concentrations, this weak quenching phenomenon persists, as the C–terminal molybdenum pterin domain of XOD lacks tryptophan and tyrosine residues. This structural feature can be clearly verified via molecular docking simulations (Figure 11A_1_). On the contrary, in the presence of chalcones, allopurinol caused more fluorescence quenching of XOD (Figure 10A_2_,A_3_). Therefore, it can be concluded that the observed fluorescence-quenching effect is mainly attributed to the contribution of butein in the complex, rather than allopurinol itself [60]. In the presence of butein, the affinity of allopurinol to (K_a_ = 1.617 × 10^5^ L·mol^−1^) was significantly higher than that of allopurinol to (K_a_ = 8.82 × 10^4^ L·mol^−1^). Similarly, allopurinol showed higher affinity (K_a_ = 1.404 × 10^5^ L·mol^−1^) in the presence of coreopsin. The results of Ka value showed that both chalcone compounds could increase the binding affinity of allopurinol to XOD, thus producing a good combination with allopurinol. In addition, the combined effect of butein and allopurinol was stronger than that of cinchonin, which was consistent with the conclusion of the ZIP synergy score.

At the same time, the effect of the synergy of butein and allopurinol on the synchronous spectrum of XOD was determined. In Supplementary Figure S4A_1_,A_2_, it was found that the fluorescence intensity of allopurinol at two Δλ was very slightly reduced, and there was basically no displacement. Compared with allopurinol alone, in the presence of butein, allopurinol caused greater fluorescence intensity reduction and blue shift at both Δλ (Supplementary Figure S4B_1_,B_2_). Such experimental observations demonstrate that co-existence with butein leads to more remarkable peak attenuation and blue shifts at both Δλ values for allopurinol–treated XOD. These synchronous fluorescence data further verify the prominent synergistic inhibitory activity of butein combined with allopurinol against XOD, and coreopsin exhibits identical trends in parallel tests (Supplementary Figure S4C_1_,C_2_).

#### 2.5.3. Allopurinol on XOD Conformation in the Presence of Chalcones

UV–vis spectra interacted with by both chalcones and allopurinol are depicted in Figure 10B_1_–B_3_. The band at 210 nm was reduced upon adding allopurinol to XOD, implying that the peptide backbone was changed by allopurinol. On the contrary, the band at 280 nm increased slowly with red shift, which suggested allopurinol could form a complex with XOD [38]. As reported by Liu et al. 2020 [21], the band change at 210 nm usually reflects peptide chain changes of XOD. As revealed in Figure 10B_2_,B_3_. Compared to allopurinol alone, in the presence of 2 chalcones, allopurinol caused a more significant decrease and red shift, which in turn led to greater conformational changes and exerted its combined effects [61].

To further understand the effect of allopurinol on XOD secondary structure in the presence of chalcones, FT–IR spectra were then scanned, and the results are shown in Figure 5B_2_. Allopurinol is a nitrogen-containing aromatic heterocyclic compound. Allopurinol exhibited a prominent band was observed at 3400 cm^−1^, attributed to N–H of aromatic ring-stretching in allopurinol. Bands at 1700 cm^−1^ came from the C–H bond and C=O bond stretching vibration. The bands (1590 cm^−1^) were made via C=C and C=N stretching vibration. The bands (1230 cm^−1^) were made via C–H stretching vibration and N–H bending vibration. In Figure 5B_2_, upon binding with allopurinol, the band in XOD shifted from 3310 to 3320 cm^− 1^, proving that hydrogen bonds were made between XOD and allopurinol [43]. This also verified thermodynamics and docking conclusions. Similarly, other peaks also showed significant shifts, such as Amide I changing from 1640 to 1700 cm^−1^, and Amide II moving from 1510 to 1530 cm^−1^. The results here confirm that XOD conformation is altered by allopurinol [62]. Compared with allopurinol, in the presence of butein and coreopsin, the Amide A band moved to 3340 and 3330 cm^−1^, Amide I shifted to 1690 and 1680 cm^−1^, and Amide II shifted to 1540 and 1530 cm^−1^, respectively. To obtain the exact secondary structure changes of XOD co-induced by chalcones and allopurinol, Amide I bands were then fitted, and the corresponding results are listed in Table 4. Compared with allopurinol alone, in the presence of 2 chalcones, allopurinol caused a greater decrease in α-helix, β-sheet, and random coil, while a greater increase in β-turn and β-antiparallel was observed. This confirmed from an FT–IR perspective that the XOD secondary structure was more altered by allopurinol in the presence of chalcones, thus proving the existence of combined effects between chalcones and allopurinol.

#### 2.5.4. The Binding Interaction Described by Multi-Molecular Docking

In order to study the binding of allopurinol to chalcone compounds, allopurinol and allopurinol–chalcone complexes were docked with XOD, respectively. In contrast to the binding sites of 2 chalcones, allopurinol entered the Mo center (Figure 11A_1_). In Figure 11A_2_, allopurinol formed hydrogen bonds with THR1010, GlU802, and ARG880, which were key amino acids in the Mo center [35,63]. In the double-docking process with two chalcones, allopurinol still interacts with XOD by forming hydrogen bonds with GLU802, ARG880, and THR1010, which is the same as the single docking results of allopurinol (Figure 11A_2_). The results confirmed that allopurinol still occupied the Mo center in the presence of chalcones [3]. In the presence of allopurinol, butein interacted with XOD by forming 8 hydrogen bonds with LEU257 (2.8 Å), GLY260 (2.1 Å), GLU263 (1.9 Å, 2.2 Å), ILE264 (2.1 Å), SER347 (1.9 Å), THR354 (2.1 Å), and ARG394 (2.0 Å) (Figure 11B_2_). Coreopsin formed 10 hydrogen bonds with VAL259 (1.8 Å, 2.7 Å), GLY260 (2.1 Å), ILE264 (1.9 Å), ALA338 (1.8 Å), SER347 (3.2 Å), ASN351 (1.9 Å), and ASP360 (1.7 Å, 2.1 Å, 2.1 Å) (Figure 11C_2_). Similar to the single docking of butein and coreopsin, two chalcones still bind to the FAD center. The above results prove that the two chalcones can bind to different sites of XOD with allopurinol, change the conformation of XOD, and have a synergistic inhibitory effect with allopurinol. The docking results further verify the results of the synergistic inhibition experiment.

## 3. Materials and Methods

### 3.1. Materials

XOD (over 0.4 units/mg protein) was from the Sigma–Aldrich Company (St. Louis, MO, USA). Xanthine, allopurinol, butein (1), coreopsin (2), okanin (3), marein (4), luteolin (5), quercetin (6), quercetagetin (7), quercetagetin–7–*O*–glucoside (8), quercetin–7–*O*–glucopyranoside (9), rutin (10), hyperin (11), naringenin (12), eriodictyol (13), isookanin (14), flavanomarein (15), and taxifolin (16) (purity > 98%) were purchased from the Chinese YuanYe Company (Shanghai, China). Other materials were bought from Chinese Solarbio Co (Beijing, China).

### 3.2. XOD Inhibitor Screening Experiments

The XOD-inhibitory potency of individual flavonoids was assessed following the procedure below. Gradient concentrations of each flavonoid were mixed with 0.04464 μmol·L^−1^ XOD and incubated at 310 K over a 15 min period. Xanthine solution (714.29 μmol·L^−1^) was then added, and the mixture was incubated for another 15 min. The absorbance of each sample (A1) was measured at 290 nm with a FlexStation–3 Microplate Reader (Molecular Devices, LLC, San Jose, CA, USA). The control sample (A3) contained XOD and xanthine, while the blank sample (A2) consisted of heat-inactivated XOD, xanthine, and flavonoid. Allopurinol served as the positive control group.

### 3.3. Radical Scavenging Experiment

In this case, flavonoids with obvious inhibitory activity on XOD were used to detect their ability to scavenge free radicals, including DPPH, O_2_^−^ produced by the non-enzymatic and enzymatic system.

#### 3.3.1. DPPH Scavenging Assay

Vitamin C served as the positive reference, and anhydrous methanol was prepared for blank control groups. A mixture consisting of 150 μL, 0.15 mmol·L^−1^ DPPH reagent and 50 μL flavonoid solutions (0–138.1 μmol·L^−1^) was incubated under dark conditions at room temperature for half an hour. After incubation, absorbance readings were recorded at 517 nm, and IC_50_ parameters were calculated according to the DPPH radical scavenging efficiency.

#### 3.3.2. Scavenging O_2_^−^ from Non-XOD System

The PMS–NADH system produces superoxide anions via a non-enzymatic reaction and has been extensively applied for evaluating antioxidant capacity. Serial concentrations of flavonoid solutions were mixed with NBT and NADH, followed by 10 min incubation at ambient temperature. The final concentrations of NBT, NADH, and flavonoids were 125 μmol·L^−1^, 780 μmol·L^−1^, and 0−138.1 μmol·L^−1^, respectively. The reaction was triggered by introducing PMS to a final concentration of 200 μmol·L^−1^. Consistent with the DPPH assay, Vitamin C served as the positive control. Absorbance values were recorded at 560 nm, and the O_2_^−^ scavenging percentage was calculated from the absorbance variations.

#### 3.3.3. O_2_^−^ Scavenging Assay by Enzymatic System

O_2_^−^ generated by XOD can convert NBT to blue formazan, which exhibited a typical OD value at 560 nm [64]. In this assay, various concentrations of flavonoids were placed in XOD solution (0.04464 μmol·L^−1^), and took incubation at 310 K (30 min). Then, 100, 200, and 500 μmol·L^−1^ of xanthine and a fixed amount of NBT were added to initiate the reaction. A blank control group was created by replacing flavonoids with a pH 7.4 PBS. OD values were indicated at 560 nm, and O_2_^−^ scavenging activity was obtained as the corresponding inhibitory ratio according to the increase in absorbance rate.

### 3.4. Combined Inhibition Screening Experiments

To further investigate flavonoids and allopurinol, flavonoids that strongly inhibit XOD, thereby inhibiting production of uric acid and O_2_^−^, were selected and screened by ZIP score using SynergyFinder (https://synergyfinder.fimm.fi, accessed on 10 September 2025). XOD was used at a concentration of 0.04 μmol·L^−1^. 1/4, 1/2, 4/5, 1, 1.5, 2 and 3 IC_50_ ratios of flavonoids/allopurinol were adopted.

### 3.5. Interaction of Chalcones with XOD and Combined with Allopurinol

Two chalcones, butein and coreopsin, exhibiting potent XOD-inhibitory activity and remarkable synergistic effects with allopurinol, were selected for further mechanistic investigation. Inhibition kinetic analysis, fluorescence spectroscopy (including synchronous and 3D fluorescence), UV–vis absorption, and FT–IR spectroscopy were employed to characterize the intermolecular interactions.

#### 3.5.1. Inhibition and Combined Kinetics Assay

The reversibility of XOD inhibition induced by chalcones was evaluated by maintaining XA at a constant concentration of 714.29 μmol·L^−1^, while simultaneously varying the concentrations of XOD and chalcones. The final concentrations of XOD were 0.00893, 0.01785, 0.02678, 0.03571, and 0.04464 μmol·L^−1^, respectively. The final concentrations of butein were 0, 1.75, 3.5, and 5.6 μmol·L^−1^, coreopsin were 0, 5.0, 8.0, 10.0 μmol·L^−1^, allopurinol were 2.0, 3.2, and 4.0 or 3.2, 4.0, and 6.0 μmol·L^−1^, respectively. To determine inhibitory type, XOD was kept as 0.04464 μmol·L^−1^, xanthine (142.86, 285.71, 428.57, 571.43, and 714.29 μmol·L^−1^), and chalcones were increased, then Lineweaver–Burk graphs were plotted. The final concentration of butein, coreopsin, and allopurinol was the same as in the reversible inhibition experiment. The incubated condition was identical to Section 2.2.

To determine the reversibility of allopurinol–butein synergistic inhibition of XOD, the concentration of XA was fixed at 714.29 μmol·L^−1^, and the concentration of butein was 3.5 μmol·L^−1^ (optimal inhibitory concentration), and allopurinol was 2.0, 3.2, and 4.0 μmol·L^−1^. Similarly, coreopsin was at 8.0 μmol·L^−1^ (optimal inhibitory concentration), and allopurinol was 3.2, 4.0, and 6.0 μmol·L^−1^. The synergistic inhibitory mode was identified with XOD concentration fixed at 0.04464 μmol·L^−1^, while synchronously raising the concentrations of XA and mixed inhibitors. XA was prepared at five gradient concentrations: 142.86, 285.71, 428.57, 571.43, and 714.29 μmol·L^−1^. The dosage gradients of chalcones and allopurinol were consistent with those adopted in the inhibition reversibility test. All incubation parameters matched the protocols described in Section 2.2.

#### 3.5.2. Fluorescence, Synchronous, and 3D Analysis

At 298, 304, 310 K, XOD (1.25 μmol·L^−1^) was put in a PBS buffer, followed by adding chalcones. Two chalcones were from 0 to 20.71 μmol·L^−1^. Fluorescence spectra of XOD upon interaction with the chalcones were recorded using an RF–7000 fluorescence spectrometer (Shimadzu Corporation, Kyoto, Japan) at an excitation wavelength of 280 nm, with emission signals scanned from 300 to 500 nm. The excitation and emission slit widths were both set to 5 nm. Meanwhile, synchronous spectra of XOD reacted with chalcones were examined at Δλ = 15 and 60 nm. 3D spectra were scanned with excitation wavelength (210–350 nm), emission wavelength (280–540 nm), and distance (2 nm).

In order to study the combined quenching mechanism of chalcones and allopurinol on XOD, allopurinol (0–33.54 μmol·L^−1^) was placed in XOD at 310 K, and the combined quenching mechanism of allopurinol on XOD was studied under the conditions of chalcones and no chalcones. In this experiment, the optimal inhibitory concentration was used (butein and coreopsin were 3.5 μmol·L^−1^ and 8.0 μmol·L^−1^). Similarly, synchronous spectra and 3D spectra were also scanned.

#### 3.5.3. UV–Vis Assay

UV–vis absorption profiles of 1.25 μmol·L^−1^ XOD were scanned at 310 K after serial supplementation of chalcones (0–20.71 μmol·L^−1^), and neat chalcone solutions were set as blank references. For synergism characterization, XOD UV spectra were collected with or without chalcones at their optimal combinatorial inhibitory doses, while allopurinol gradients spanning 0 to 33.54 μmol·L^−1^ were introduced. An Agilent Cary–300 UV–vis spectrophotometer (Agilent Technologies, Inc., Santa Clara, CA, USA) was employed for all spectral scans across the 200–450 nm wavelength region.

#### 3.5.4. FT–IR Assay

All samples containing isolated XOD or XOD–chalcone binding complexes were frozen at −80 °C and lyophilized, then subjected to FT–IR measurement via a Nicolet iS5 spectrometer (Thermo Fisher Scientific, Inc., Madison, WI, USA). Each mixture was formulated with 1.25 μmol·L^−1^ XOD, 20.71 μmol·L^−1^ chalcones, and 33.54 μmol·L^−1^ allopurinol. KBr powder was adopted for background correction, and full-range spectra were scanned across the wavenumber region of 600–4000 cm^−1^. The Amide I spectral region spanning 1600–1700 cm^−1^ was processed with PeakFit 4.12 to analyze conformational changes in XOD.

### 3.6. Molecular Dynamics Simulation

XOD structure (1FIQ) was selected using https://www.rcsb.org/ (accessed on 10 September 2025), the H_2_O molecular in XOD was deleted, an H atom was added by Pymol (v3.1.0a0), and the pdb was output. Structures of butein (CID5281222), coreopsin (CID12303943), and allopurinol (CID135401907) were obtained from PubChem, then hydrogenation, charge adjustment, and ligand distribution were made. XOD acted as a rigid protein; coreopsin, butein, and allopurinol served as flexible ligands. To verify the combined effect of chalcones and allopurinol on XOD, chalcones and allopurinol were docked with XOD simultaneously. The calculation space was defined by a 100 × 100 × 100 Å grid box with an interval of 0.525 Å between adjacent grid points. Following 100 rounds of molecular docking calculations, the obtained output data were processed and visualized using the Lamarckian genetic algorithm, Pymol, and Discovery Studio 2019.

Molecular dynamics (MD) simulations were carried out via GROMACS 2020.6 to track the conformational evolution of XOD upon binding two chalcones and allopurinol. Three independent systems (free XOD, XOD–chalcone binary complexes, XOD–allopurinol complex) were separately solvated within cubic water-filled simulation boxes. Sodium and chloride ions were supplemented to neutralize residual surface electrostatic charges of protein–ligand assemblies. The steepest descent algorithm was adopted for full-system energy minimization. Subsequent equilibration stages contained a 50,000-step NVT ensemble at 310 K, followed by a 100 ps NPT run under 1 bar constant pressure. After a 100 ns production MD trajectory, structural metrics including RMSD, RMSF, SASA, and Rg were extracted for quantitative structural analysis.

### 3.7. MM–PBSA Calculation and Independent-Gradient Model Analysis

MM–PBSA calculations were implemented to quantify binding free energies, to evaluate the aqueous binding stability of XOD with the two chalcone compounds. The Multiwfn program, combined with IGM, was adopted to dissect intermolecular contacts between chalcones and XOD, while VMD software (v1.9.4) was utilized for visualization of representative molecular conformations.

### 3.8. Statistical Analysis

Triplicate parallel tests were set up for all experimental groups, with final outcomes presented in the form of mean ± SD. One-way ANOVA combined with the Duncan test was adopted for data processing using GraphPad Prism 8. Statistical significance was determined at the threshold of *p* < 0.05.

## 4. Conclusions

In conclusion, 8 flavonoids were screened as potential XOD inhibitors. Among them, butein and coreopsin showed strong inhibition of XOD because they inhibited not only uric acid but also O_2_^−^ produced by XOD. Interestingly, 2 chalcones revealed strong combined effects with allopurinol by multispectral experiments. The molecular docking results show that 2 chalcones could bind to the FAD center through hydrogen bonds and van der Waals forces, forming stable complexes, inducing conformational changes, and thereby exerting inhibitory effects. Some crucial amino acids provided more electrostatic and van der Waals energies to participate in maintaining complex stability. Then, intermolecular interactions of 2 chalcones with XOD were visualized using IGM. Two chalcones could bind to different sites with allopurinol, change XOD conformation, and play a combined inhibitory role with allopurinol. Based on the in vitro tests and computer simulations herein, butein and coreopsin might potentially act synergistically with allopurinol against XOD. Since only in vitro measurements were performed, follow-up cellular and animal studies are needed to validate these findings.

## Figures and Tables

**Table 1:** Chemical structure of 16 flavonoids.

Type	Name	Substitutions	
		OH	Glycosyl
Chalcone	Butein	7,9,3′,4′	
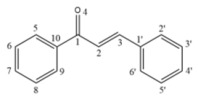	Coreopsin	5,4′,5′	7-*O*-glucopyranoside
	Okanin	7,8,9,3′,4′	
	Marein	5,6,3′,4′	7-*O*-glucopyranoside
Flavones	luteolin	5,7,3′,4′	
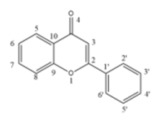	Quercetin	3,5,7,3′,4′	
	Quercetagetin	3,5,6,7,3′,4′,	
	Quercetagetin-7-*O*-glucoside	3,5,6,3′,4′,	7-*O*-glucopyranoside
	Quercetin-7-*O*-glucopyranoside	3,5,3′4′	7-*O*-glucopyranoside
	Rutin	5,7,3′,4′	3-*O*-rhamnosylglucoside
	Hyperin	5,7,3′,4′	3-*O*-galactoside
Flavanon	Naringenin	5,7,4′	
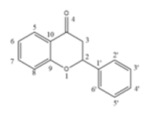	Eriodictyol	5,7,3′,4′	
	Isookanin	7,8,3′,4′	
	Flavanomarein	8,3′,4′	7-*O*-glucopyranoside
	Taxifolin	3,5,7,3′4′	

**Table 2:** XOD inhibition and free-radical scavenging data.

Name	IC_50_			Scavenging Activity (%)	ZIP Score
	XOD	DPPH	O_2_^−^ Produced by XOD	O_2_^−^ in Non-Enzyme Catalytic	
Allopurinol	4.16 ± 0.1	–	–	–	
Butein	7.56 ± 0.2	8.00 ± 0.3	8.79 ± 0.5	23.00 ± 0.2	18.19 ± 0.4
Coreopsin	10.43 ± 0.4	10.42 ± 0.2	13.38 ± 0.4	17.64 ± 0.3	15.52 ± 0.4
Okanin	17.56 ± 0.2	20.33 ± 0.4	11.99 ± 0.2	22.58 ± 0.4	6.54 ± 0.5
luteolin	6.95 ± 0.3	8.43 ± 0.5	12.15 ± 0.1	45.75 ± 0.5	8.86 ± 0.2
Quercetin	8.60 ± 0.3	10.84 ± 0.1	5.42 ± 0.3	22.36 ± 0.2	3.80 ± 0.4
Quercetin-7-*O*-glucopyranoside	83.52 ± 0.6	65.70 ± 0.2	76.78 ± 0.6	39.19 ± 0.3	1.03 ± 0.2
Rutin	86.26 ± 0.5	72.93 ± 0.6	72.53 ± 0.2	35.32 ± 0.5	−3.40 ± 0.1
Hyperin	30.25 ± 0.2	41.52 ± 0.5	24.64 ± 0.1	37.18 ± 0.3	0.27 ± 0.3

**Table 3:** K_SV_, K_q_, K_b_, ΔG, ΔH, and ΔS of XOD–chalcone.

Complex	T (K)	K_SV_ (×10^4^ L·mol^−1^)	K_q_ (×10^12^ L·mol^−1^·s^−1^)	K_a_ (×10^4^ L·mol^−1^)	ΔG (kJ·mol^−1^)	ΔH (kJ·mol^−1^)	ΔS (J·mol^−1^ K^−1^)
XOD–butein	298	5.69 ± 0.02	5.69 ± 0.02	12.22 ± 0.06	−29.70 ± 0.01	−71.00 ± 0.10	−138.59 ± 0.08
	304	4.71 ± 0.01	4.71 ± 0.04	9.77 ± 0.02	−28.87 ± 0.06		
	310	4.16 ± 0.02	4.16 ± 0.01	5.02 ± 0.01	−28.03 ± 0.01		
XOD–coreopsin	298	9.75 ± 0.01	9.75 ± 0.02	11.64 ± 0.05	−28.89 ± 0.05	−60.75 ± 0.07	−106.92 ± 0.10
	304	8.81 ± 0.02	8.81 ± 0.03	6.39 ± 0.01	−28.25 ± 0.01		
	310	6.70 ± 0.03	6.70 ± 0.01	4.51 ± 0.01	−27.61 ± 0.02		

**Table 4:** Secondary structure contents of XOD with or without butein/coreopsin/allopurinol.

Complex	α–Helix (%)	β–Sheet (%)	β–Turn (%)	Random Coil (%)	β–Antiparallel (%)
XOD	24.39 ± 0.02	46.22 ± 0.03	11.56 ± 0.01	16.61 ± 0.01	1.21 ± 0.03
XOD–butein	22.24 ± 0.03	41.32 ± 0.02	10.65 ± 0.05	23.71 ± 0.05	2.08 ± 0.02
XOD–coreopsin	22.86 ± 0.02	42.67 ± 0.06	9.94 ± 0.02	21.18 ± 0.02	1.95 ± 0.02
XOD–allopurinol	21.11 ± 0.05	40.43 ± 0.01	10.58 ± 0.05	25.32 ± 0.04	2.55 ± 0.03
XOD–allopurinol–butein	20.91 ± 0.03	37.71 ± 0.03	10.88 ± 0.02	24.71 ± 0.03	5.78 ± 0.02
XOD–allopurinol–coreopsin	20.86 ± 0.02	38.91 ± 0.04	10.80 ± 0.04	24.47 ± 0.01	4.96 ± 0.04

## Data Availability

Data will be made available on request.

## Supplementary Materials

The following supporting information can be downloaded at: https://www.mdpi.com/article/10.3390/molecules31142485/s1, Figure S1: (A–E) Inhibition effects of flavonoids 3, 5, 9, 10 and 11 on O_2_^−^ generated by XOD at different [xanthine]; Figure S2: Heat map and ZIP synergy score of 8 flavonoids; Figure S3: (A_1–3_) Synchronous spectra and RSFQ of XOD induced by butein and (B_1–3_) coreopsin. (C_1–3_) 3D spectra of XOD, XOD–butein and XOD–coreopsin; Figure S4: Synchronous spectra and RSFQ of XOD induced by allopurinol (A_1–3_), butein–allopurinol (B_1–3_), and coreopsin–allopurinol (C_1–3_). 3D spectra of XOD, XOD–allopurinol, XOD–butein–allopurinol and XOD–coreopsin–allopurinol.

## References

[1] Zeng N., Zhang G., Hu X., Pan J., Gong D. (2019). Mechanism of Fisetin Suppressing Superoxide Anion and Xanthine Oxidase Activity. J. Funct. Foods.

[2] Yang Y.-S., Wang B., Liu J., Li Q., Jiao Q.-C., Qin P. (2023). Discovery of Coumaric Acid Derivatives Hinted by Coastal Marine Source to Seek for Uric Acid Lowering Agents. J. Enzym. Inhib. Med. Chem..

[3] Wen J., Chen S., Deng L., Chen X., Jiang X., Hu X., Gong D., Zhang G. (2024). Inhibitory Mechanism of Phloretin on Xanthine Oxidase and Its Synergistic Effect with Allopurinol and Febuxostat. Food Biosci..

[4] Masuoka N. (2021). Stilbene Compounds Are Specific Inhibitors of the Superoxide Anion Generation Catalyzed by Xanthine Oxidase. Food Chem. X.

[5] Zhang C., Zhang G., Pan J., Gong D. (2016). Galangin Competitively Inhibits Xanthine Oxidase by a Ping-Pong Mechanism. Food Res. Int..

[6] Zhang G., Zhu M., Liao Y., Gong D., Hu X. (2022). Action Mechanisms of Two Key Xanthine Oxidase Inhibitors in Tea Polyphenols and Their Combined Effect with Allopurinol. J. Sci. Food Agric..

[7] Zhao J., Huang L., Sun C., Zhao D., Tang H. (2020). Studies on the Structure-Activity Relationship and Interaction Mechanism of Flavonoids and Xanthine Oxidase through Enzyme Kinetics, Spectroscopy Methods and Molecular Simulations. Food Chem..

[8] Zeng N., Zhang G., Hu X., Pan J., Zhou Z., Gong D. (2018). Inhibition Mechanism of Baicalein and Baicalin on Xanthine Oxidase and Their Synergistic Effect with Allopurinol. J. Funct. Foods.

[9] Guo L., Yang C., Yang R., Zhao W. (2021). Magnetically Anchored Antibody-Coupled Nanocomposite as α-Amylase Inhibitor for Long-Time Protection against Glycemic Variability. Chem. Eng. J..

[10] Guo L., Zhang W., Li S., Ho C.-T. (2015). Chemical and Nutraceutical Properties of Coreopsis Tinctoria. J. Funct. Foods.

[11] Lin S., Zhang G., Liao Y., Pan J., Gong D. (2015). Dietary Flavonoids as Xanthine Oxidase Inhibitors: Structure–Affinity and Structure–Activity Relationships. J. Agric. Food Chem..

[12] Li K., Wang Y., Liu W., Zhang C., Xi Y., Zhou Y., Li H., Liu X. (2024). Structure–Activity Relationships and Changes in the Inhibition of Xanthine Oxidase by Polyphenols: A Review. Foods.

[13] Xue H., Xu M., Gong D., Zhang G. (2023). Mechanism of Flavonoids Inhibiting Xanthine Oxidase and Alleviating Hyperuricemia from Structure–Activity Relationship and Animal Experiments: A Review. Food Front..

[14] Hou Y.-X., Sun S., Yang L., Li Y., Liu X., Wang W., Zhang S. (2019). An Improved Method for the Synthesis of Butein Using SOCl_2_/EtOH as Catalyst and Deciphering Its Inhibition Mechanism on Xanthine Oxidase. Molecules.

[15] Xiao H., Wang H., Sun S., Ma J., Wang H., Yin Y., Wang W. (2024). Identification of Aurones and Chalcones as the Main Contributors to Xanthine Oxidase Inhibitory Activity of Snow Chrysanthemum. Food Biosci..

[16] Tey Z.T., Loh K.E., Tan S.-P., Yuan C., Tejo B.A., Ismail I.S., bin Nafiah M.A. (2024). Xanthine Oxidase Inhibitory Activity by Flavonoids from *Chrysanthemum morifolium*: In Vitro and in Silico Insights. Phytochem. Lett..

[17] Liu Y., Yu D., Liu Y., Li L., Yang C., Chen H., Li Y. (2025). Investigation of Binding Mechanism for Newly Synthesized Apigenin Derivatives with Xanthine Oxidase: Spectroscopic and Computational Approach. J. Mol. Struct..

[18] Sharma O.P., Bhat T.K. (2009). DPPH Antioxidant Assay Revisited. Food Chem..

[19] Zhu M., Pan J., Hu X., Zhang G. (2021). Epicatechin Gallate as Xanthine Oxidase Inhibitor: Inhibitory Kinetics, Binding Characteristics, Synergistic Inhibition, and Action Mechanism. Foods.

[20] Rayene K., Imane D., Abdelaziz B., Leila N., Fatiha M., Abdelkrim G., Bouzid G., Ismahan L., Brahim H., Rabah O. (2021). Molecular Modeling Study of Structures, Hirschfield Surface, NBO, AIM, RDG, IGM and 1H NMR of Thymoquinone/Hydroxypropyl-β-Cyclodextrin Inclusion Complex from QM Calculations. J. Mol. Struct..

[21] Liu Y., Chen H., Xiang H., Lei H., Zhang D., Qiu Y., Xu L. (2020). Inhibition and Molecular Mechanism of Diosmetin against Xanthine Oxidase by Multiple Spectroscopies and Molecular Docking. New J. Chem..

[22] Liu X., Zhang W., Chen J., Fu R., Lin X., Zhou S., Wang L. (2024). Investigating the Inhibition of Xanthine Oxidase by Five Catechins: Kinetic Studies, Spectroscopy, Molecular Docking, and Dynamics Simulations. Int. J. Biol. Macromol..

[23] Chen R., Shi Y., Liu G., Tao Y., Fan Y., Wang X., Li L. (2021). Spectroscopic Studies and Molecular Docking on the Interaction of Delphinidin-3-*O*-Galactoside with Tyrosinase. Biotechnol. Appl. Biochem..

[24] Luo Y., Xie J., Yang H., Huang Y., Yang X. (2025). Xanthine Oxidase Inhibitory and Anti-Hyperuricemic Properties of Sulfonate Derivatives of Naringenin. Bioorg. Chem..

[25] Wang R., Guo P.-F., Nie J., Hu X., Wu Y.-W., Zhu S., Chen B.-R., Li J., Zeng X.-A., Xu F.-Y. (2024). Inhibitory Mechanism of Xanthine Oxidase by 6-, 8- and 10-Gingerol: Enzyme Kinetics, Multi-Spectroscopy and Molecular Simulations. J. Mol. Liq..

[26] Song J., Wang Z., Chi Y., Zhang Y., Fang C., Shu Y., Cui J., Bai H., Wang J. (2023). Anti-Gout Activity and the Interaction Mechanisms between Sanghuangporus Vaninii Active Components and Xanthine Oxidase. Bioorg. Chem..

[27] Yang Y., Chen Q., Ruan S., Ao J., Liao S.-G. (2022). Insights into the Inhibitory Mechanism of Viniferifuran on Xanthine Oxidase by Multiple Spectroscopic Techniques and Molecular Docking. Molecules.

[28] Zhang X., Qu Z., Cui Y., Du Y., Li Y., Li L. (2026). Intermolecular Interaction of Coreopsin with β-Lactoglobulin at Varied pH Values: Experiment, Dynamics Simulation and IGM Analysis. Food Chem..

[29] Chen J., Wang Y., Pan X., Cheng Y., Liu J., Cao X. (2022). Study on the Interaction Mechanism between Luteoloside and Xanthine Oxidase by Multi-Spectroscopic and Molecular Docking Methods. J. Mol. Recognit..

[30] Yao Y., Zhang M., Chen J., Ma A., Huang Q., Yan J., Yang H., Chen S. (2025). Novel Mechanistic Insights into Luteolin and Its Analogues as Potent Inhibitors of Xanthine Oxidase Activity: Inhibition Thermodynamics, Kinetics and Docking Simulation. Food Biosci..

[31] Wan Y., Qian J., Li Y., Shen Y., Chen Y., Fu G., Xie M. (2021). Inhibitory Mechanism of Xanthine Oxidase Activity by Caffeoylquinic Acids in Vitro. Int. J. Biol. Macromol..

[32] Wang J., Sun S., Zhao K., Shi H., Fan J., Wang H., Liu Y., Liu X., Wang W. (2020). Insights into the Inhibitory Mechanism of Purpurogallin on Xanthine Oxidase by Multiple Spectroscopic Techniques and Molecular Docking. J. Mol. Struct..

[33] Li M., Li C., Tao T., Li W., Kang M., Fu P., Liu X., Quan W., Zhu M., Liu Z. (2025). Study on the Synergistical Effects of Characteristic Compounds in Osmanthus Black Tea against Xanthine Oxidase Based on Multispectral Analysis Combined with in Silico Studies. Int. J. Biol. Macromol..

[34] Yang C., Xu H., Zhao P., Yin Q., Wang N., Li L., Liu Y., Li Y. (2025). Molecular Insights from Multi-Spectroscopy and Computer Simulations Investigation on the Xanthine Oxidase Inhibition by 2′,4′-Dimethoxy-4-Hydroxychalcone. Bioorg. Chem..

[35] Yan X., Feng B., Song H., Wang L., Wang Y., Sun Y., Cai X., Rong Y., Wang X., Wang Y. (2025). Identification and Mechanistic Study of Piceatannol as a Natural Xanthine Oxidase Inhibitor. Int. J. Biol. Macromol..

[36] He P., Xu H., Yang C., Yu D., Liu Y., Du J., Li Y. (2023). Unveiling the Inhibitory Mechanism of Aureusidin Targeting Xanthine Oxidase by Multi-Spectroscopic Methods and Molecular Simulations. RSC Adv..

[37] Liu Y., Han C., Lu T., Liu Y., Chen H., Yang C., Tu Y., Li Y. (2021). Investigation of the Interaction between Chrysoeriol and Xanthine Oxidase Using Computational and in Vitro Approaches. Int. J. Biol. Macromol..

[38] Zhu M., Wang L., Wang Y., Zhou J., Ding J., Li W., Xin Y., Fan S., Wang Z., Wang Y. (2018). Biointeractions of Herbicide Atrazine with Human Serum Albumin: UV-Vis, Fluorescence and Circular Dichroism Approaches. Int. J. Environ. Res. Public Health.

[39] Paczkowska M., Lewandowska K., Bednarski W., Mizera M., Podborska A., Krause K., Cielecka-Piontek J. (2015). Application of Spectroscopic Methods for Identification (FT-IR, Raman Spectroscopy) and Determination (UV, EPR) of Quercetin-3-*O*-Rutinoside. Experimental and DFT Based Approach. Spectrochim. Acta Part A.

[40] Xie J., Yang F., Shi H., Yan J., Shen H., Yu S., Gan N., Feng B., Wang L. (2022). Protein FT-IR Amide Bands Are Beneficial to Bacterial Typing. Int. J. Biol. Macromol..

[41] Tang C., Lin K., Zhou X., Liu S. (2016). In Situ Detection of Amide a Bands of Proteins in Water by Raman Ratio Spectrum. Chin. J. Chem. Phys..

[42] Han L., Zhang K., Ishida H., Froimowicz P. (2017). Study of the Effects of Intramolecular and Intermolecular Hydrogen-Bonding Systems on the Polymerization of Amide-Containing Benzoxazines. Macromol. Chem. Phys..

[43] Li T., Wang L., Chen Z., Zhang X., Zhu Z. (2020). Functional Properties and Structural Changes of Rice Proteins with Anthocyanins Complexation. Food Chem..

[44] Lin D., Li N., Wei C., Wang Z. (2023). The Changes in Molecular Interaction and Conformation of Lysozyme Mediated by Ionic Liquids. J. Mol. Liq..

[45] Song X., Ni M., Zhang Y., Zhang G., Pan J., Gong D. (2021). Comparing the Inhibitory Abilities of Epigallocatechin-3-Gallate and Gallocatechin Gallate against Tyrosinase and Their Combined Effects with Kojic Acid. Food Chem..

[46] Zhang C., Wang R., Zhang G., Gong D. (2018). Mechanistic Insights into the Inhibition of Quercetin on Xanthine Oxidase. Int. J. Biol. Macromol..

[47] Wang X., Su D., Luo X., Chen B., Bhullar K.S., Liu H., Wang C., Zhang J., Wang L., Yang H. (2025). Inhibition of Xanthine Oxidase by Four Phenolic Acids: Kinetic, Spectroscopic, Molecular Simulation, and Cellular Insights. Foods.

[48] Hicham A., Amal B., Amina B., Said G. (2024). In Silico Study of the Xanthine Oxidase Enzyme Inhibition by the Essential Oil Compounds of Myrtus Communis Plant in the Treatment of Gout Disease. React. Kinet. Mech. Catal..

[49] Xie C., Zhai N., Zheng X., Jiang M., Zhao Y., Xu Z., Sun Q., Liu G. (2024). Exploration of Novel Non-Purine Xanthine Oxidase Inhibitors Based on Oxadiazolones by an Integrated Simulation Study. New J. Chem..

[50] Zhu Y.-X., Sheng Y.-J., Ma Y.-Q., Ding H.-M. (2022). Assessing the Performance of Screening MM/PBSA in Protein–Ligand Interactions. J. Phys. Chem. B.

[51] Nguyen A.H., Do A.T., Linh N.N., Pham M.Q., Ngo S.T., Le T.-K.-D. (2026). Searching for Potential Xanthine Oxidase Inhibitors from Vietnamese Herb Plants via Computational Studies. Comput. Biol. Chem..

[52] Martins L.C., Torres P.H.M., de Oliveira R.B., Pascutti P.G., Cino E.A., Ferreira R.S. (2018). Investigation of the Binding Mode of a Novel Cruzain Inhibitor by Docking, Molecular Dynamics, Ab Initio and MM/PBSA Calculations. J. Comput.-Aided Mol. Des..

[53] Varughese J.K., Jose J., Devi P.R., Ali N., Parvez M.K., Al-Dosari M.S., Nishat S., Mathew T.V. (2025). Inhibitory Activity of Chalcones as MAO-B Inhibitors: An Insight from Molecular Docking, ADME Analysis, MD Simulation, and MM-PBSA Calculations. ChemistrySelect.

[54] Lu T., Chen Q. (2022). Independent Gradient Model Based on Hirshfeld Partition: A New Method for Visual Study of Interactions in Chemical Systems. J. Comput. Chem..

[55] Lefebvre C., Klein J., Khartabil H., Boisson J.-C., Hénon E. (2023). IGMPlot: A Program to Identify, Characterize, and Quantify Molecular Interactions. J. Comput. Chem..

[56] Benaïssa A., Bouhadiba A., Naili N., Chekkal F., Khelfaoui M., Bouras I., Madjram M.S., Zouchoune B., Mogalli S., Malfi N. (2023). Computational Investigation of Dimethoate and β-Cyclodextrin Inclusion Complex: Molecular Structures, Intermolecular Interactions, and Electronic Analysis. Struct. Chem..

[57] Yang M., Huang D., Wu H., Zhang H., An P., Yuan C., Su P., Luo Z. (2019). Unravelling the Weak Interactions in Binary Clusters of Serotonin and Amino Acid Residues. Chem. Sel..

[58] Petrushenko I.K., Saha B. (2025). A Theoretical Study on the Interaction of Orthosilicic Acid with Water Molecules. Chem. Phys..

[59] Ni J., Wen X., Wang S., Zhou X., Wang H. (2023). Investigation of the Inhibitory Combined Effect and Mechanism of (−)-Epigallocatechin Gallate and Chlorogenic Acid on Amylase: Evidence of Synergistic Interaction. Food Biosci..

[60] Yao Y., Ye J., Chen L., Ma A., Pang X., Song S., Zhang M., Chen S., Yang H. (2026). Deciphering the Synergistic Inhibition Mechanism of Xanthine Oxidase by Myricetin and Allopurinol: Insights from Inhibition Kinetics, Thermodynamics, and Molecular Dynamic Simulations. Bioorg. Chem..

[61] Vijeesh V., Jisha N., Vysakh A., Latha M.S. (2021). Interaction of Eugenol with Xanthine Oxidase: Multi Spectroscopic and in Silico Modelling Approach. Spectrochim. Acta Part A.

[62] Chatterley A.S., Laity P., Holland C., Weidner T., Woutersen S., Giubertoni G. (2022). Broadband Multidimensional Spectroscopy Identifies the Amide II Vibrations in Silkworm Films. Molecules.

[63] Ghallab D.S., Shawky E., Metwally A.M., Celik I., Ibrahim R.S., Mohyeldin M.M. (2022). Integrated in Silico—In Vitro Strategy for the Discovery of Potential Xanthine Oxidase Inhibitors from Egyptian Propolis and Their Synergistic Effect with Allopurinol and Febuxostat. RSC Adv..

[64] Polovinkina M.A., Osipova V.P., Osipova A.D., Berberova N.T., Velikorodov A.V., Matishov G.G. (2021). Activity of Hydroxy Derivatives of Chalcones toward Superoxide Anion Radical. Dokl. Chem..

