# Supplementary material for: Screening and Mechanistic Investigation of Synergistic Xanthine Oxidase Inhibitors Based on Structure–Activity Relationships
Source: Molecules. 2026 Jul 16;31(14):2485. doi: 10.3390/molecules31142485 (PMC13414348; doi:10.3390/molecules31142485)

Figure S1 (A-E) Inhibition effects of flavonoids 3, 5, 9, 10 and 11 on O_2^- generated by XOD at different [xanthine].

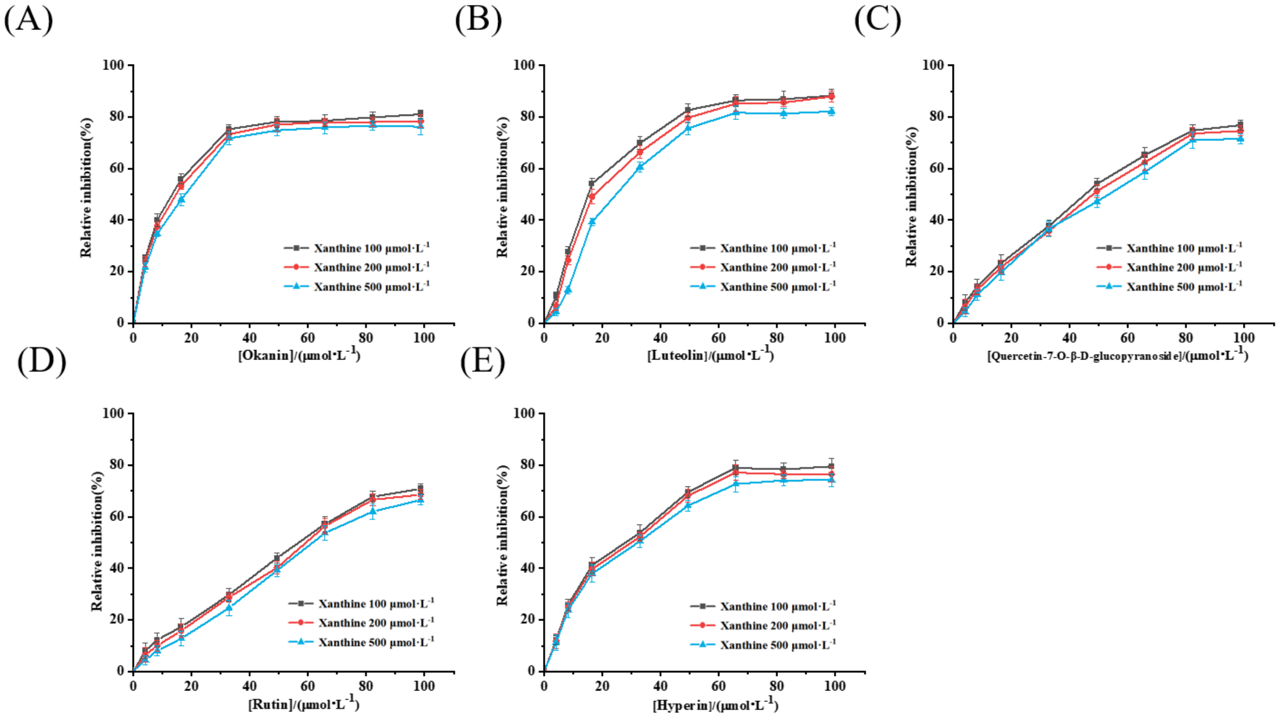

Figure S2 Heat map and ZIP synergy score of 8 flavonoids.

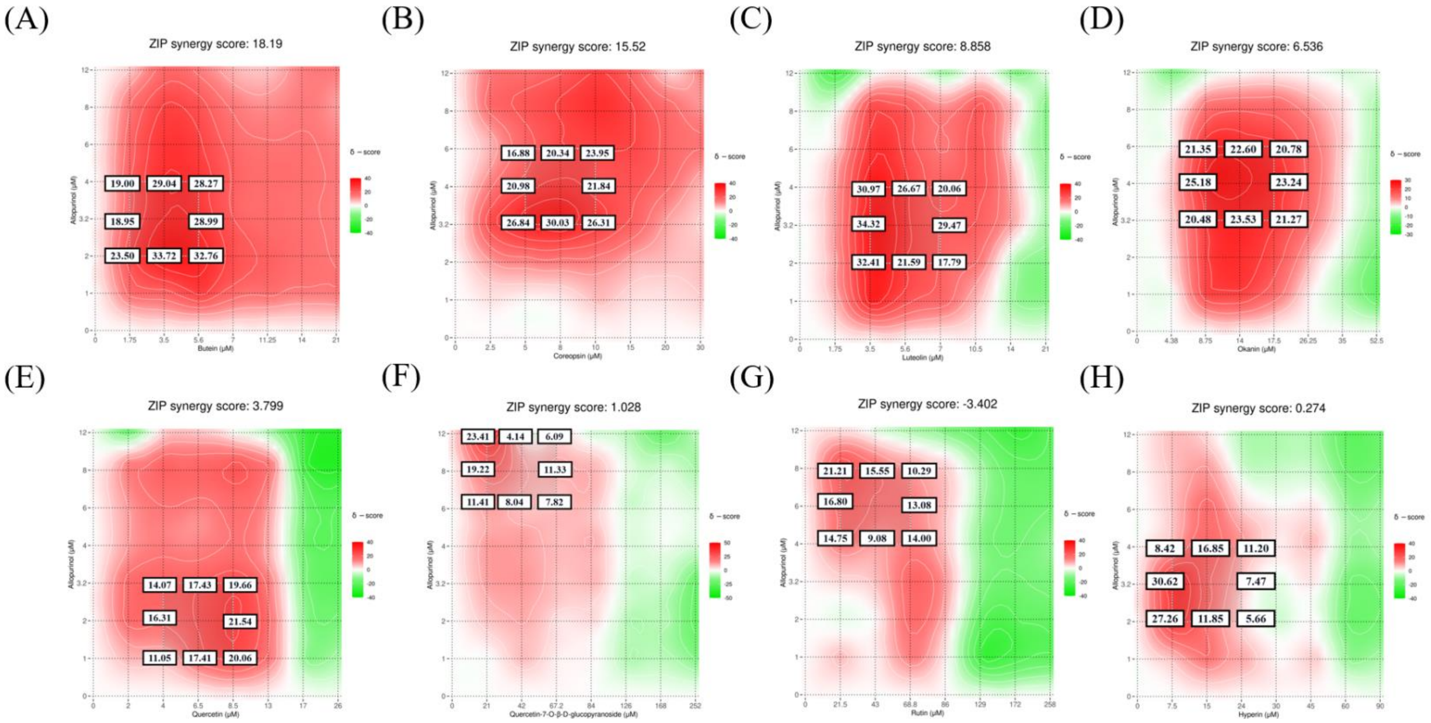

Figure S3 (A_{1-3}) Synchronous spectra and RSFQ of XOD induced by butein and (B_{1-3}) coreopsin. (C_{1-3}) 3D spectra of XOD, XOD−butein and XOD−coreopsin.

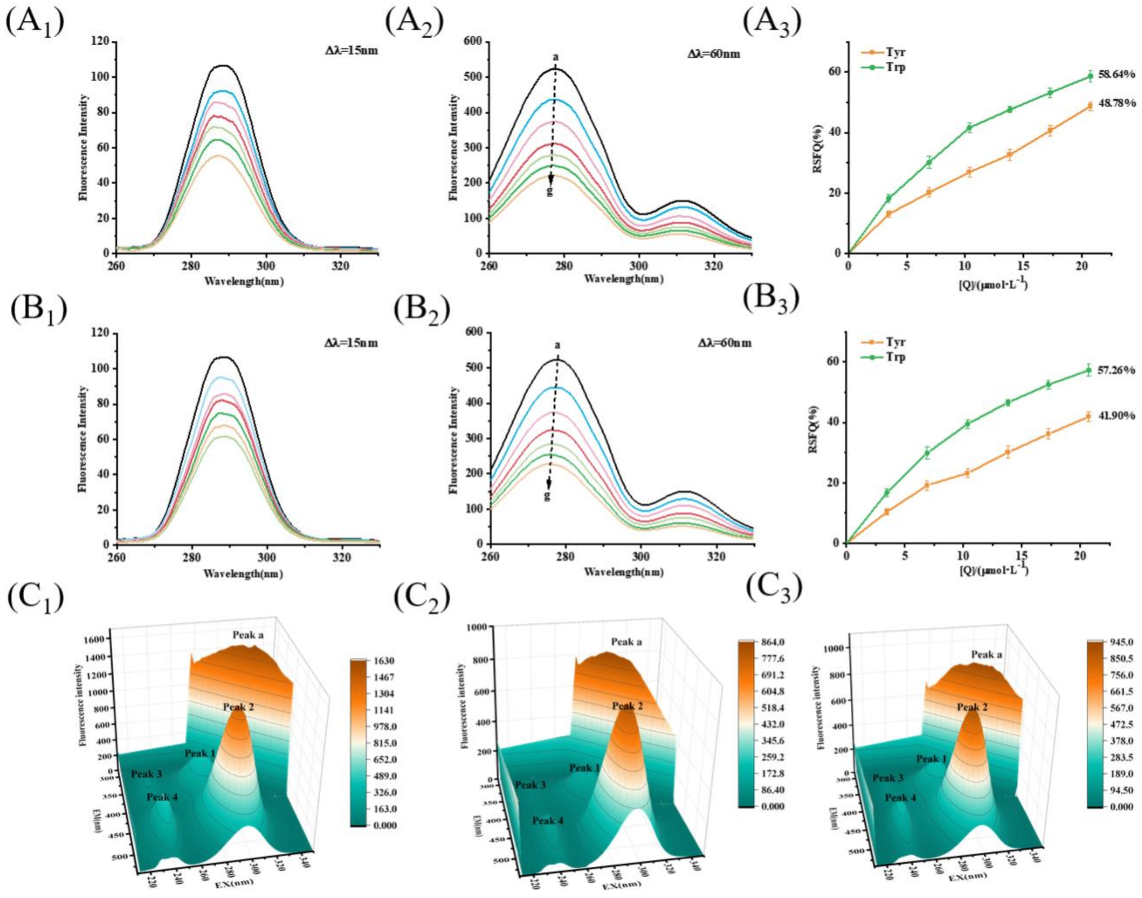

Figure S4 Synchronous spectra and RSFQ of XOD induced by allopurinol (A_{1-3}), butein-allopurinol (B_{1-3}) and coreopsin-allopurinol (C_{1-3}). 3D spectra of XOD, XOD-allopurinol, XOD-butein-allopurinol and XOD-coreopsin-allopurinol.

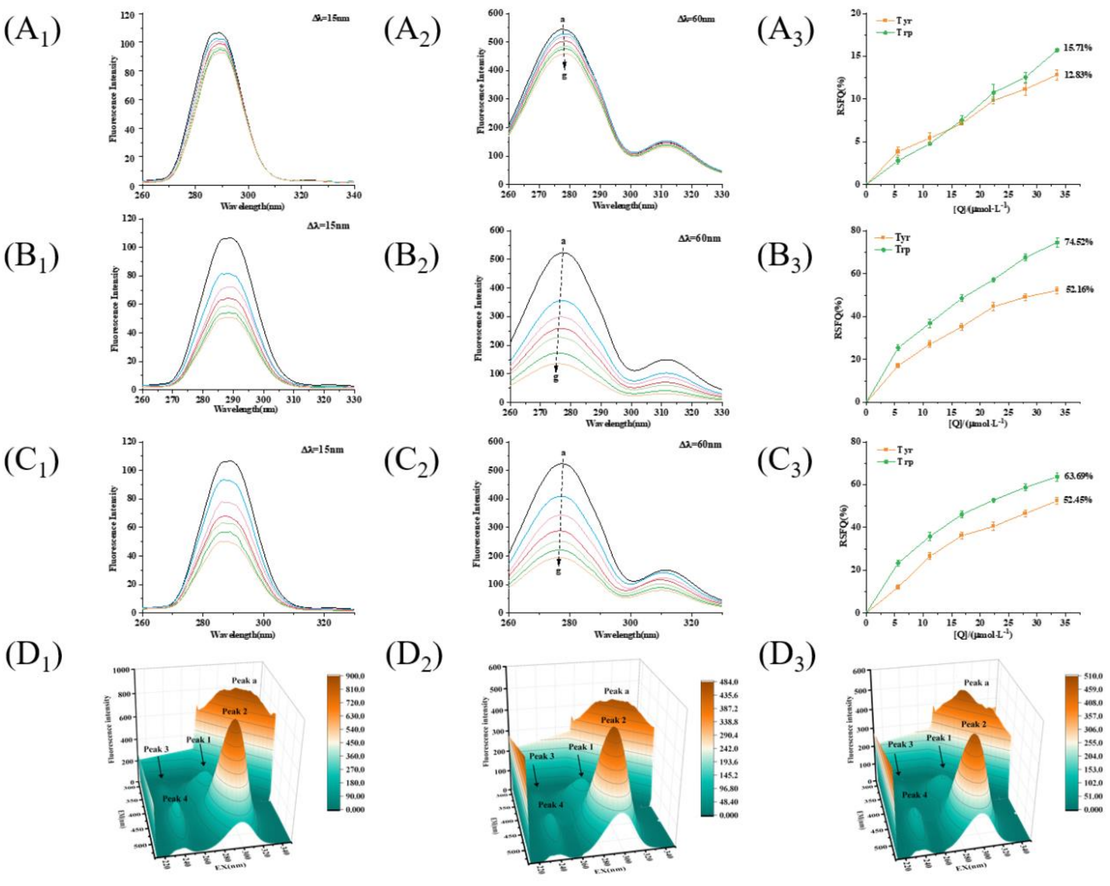

Supplement: Supplementary file 1 [file molecules-31-02485-s001.zip › molecules-4421409-supplementary.pdf]
